# Electronic properties and optical spectra of donor–acceptor conjugated organic polymers

**DOI:** 10.1038/s41598-023-48468-9

**Published:** 2023-12-07

**Authors:** Chandra Shekar Sarap, Yashpal Singh, John Michael Lane, Neeraj Rai

**Affiliations:** Dave C. Swalm School of Chemical Engineering and Center for Advanced Vehicular Systems, Mississippi State University, Mississippi State, MS 39762 USA

**Keywords:** Chemistry, Theoretical chemistry, Computational chemistry, Materials science, Theory and computation, Electronic structure

## Abstract

Organic semiconductors based on conjugated donor-acceptor (D–A) polymers are a unique platform for electronic, spintronic, and energy-harvesting devices. Understanding the electronic structure of D–A polymers with a small band gap is essential for developing next-generation technologies. Here, we investigate the electronic structure and optical spectra of cyclopentadithiophene-based closed/open-shell D–A polymers using density functional theory and the Bethe–Salpeter equation based on G$_0$W$_0$ approximation. We explored the role of different acceptor units and chemical substitutions on the structural changes and, more importantly, electronic, optical, and dielectric behavior. We found that the computed first exciton peak of the polymers agreed well with the available experimentally measured optical gap. Furthermore, D–A polymers with open-shell character display higher dielectric constant than the closed-shell polymers. We show that the exceptional performance of polycyclopentadithiophene-thiophenylthiadiazoloquinoxaline (PCPDT-TTQ) as a scalable *n*-type material for Faradaic supercapacitors can be partly ascribed to its elevated dielectric constant. Consequently, these D–A polymers, characterized by their high dielectric constants, exhibit significant potential for various applications, including energy storage, organic electronics, and the production of dielectric films.

## Introduction

Organic electronic materials comprise a broad range of small molecules to polymers with versatile optoelectronic properties. These materials are used in different applications, including supercapacitors, organic light-emitting diodes, photovoltaics, biosensor, and storage devices^1–7^. Moreover, electronic materials exhibiting open-shell character and high-spin ground state can accelerate the development of spintronics or magneto-electronic devices for quantum computing applications^4,8–11^. Among various organic materials, donor–acceptor (D–A) polymers are among the most successful approaches to achieving tunable bandgap, including high-spin states^12–14^. These polymers can lead to efficient inter- and intra-molecular charge transfer as the exciton migration benefits from the $\pi$-stacking due to film crystallinity and exhibits efficient charge transport^15^. However, the intrinsic electronic structure properties of the open-shell polymers are distinctive and depend on the nature of the donor and acceptor groups. As the interaction of light with this complex donor-acceptor polymers is at the heart of many photonic devices, it is essential to investigate their optical spectrum using advanced many-body methods.

Accurately computing the optical spectra of organic polymers is a challenging task, especially for complex systems such as amorphous or semi-crystalline D–A polymers. Current methods are limited to closed-shell systems such as polyethylene, polypropylene, polyterephthalate, polythiophene, and linear polymers/oligomers of D–A systems^16–21^. Although density functional theory (DFT) can describe the electronic structure and bandgap of these polymers, the accuracy of the excitonic properties is insufficient, and calculations are often restricted to small molecules/oligomeric units^22–25^. Molecular solids have been studied extensively using DFT and high-level *ab initio* methods, such as many-body perturbation-based GW (G: Greens function, W: Screened-Coulomb interaction) approximation^26–28^. Time-dependent DFT (TDDFT) with range-separated hybrid functional can provide better predictions of the optical absorption for small organic molecular solids, similar to the Bethe–Salpeter equation (BSE) based on the G$_0$W$_0$ method (also known as single-shot GW approximation) and experiments^27^. However, extending the above methods to polymers is computationally expensive but required for developing a better understanding of the electronic structure and optical properties of D–A polymers.

**Figure 1:**
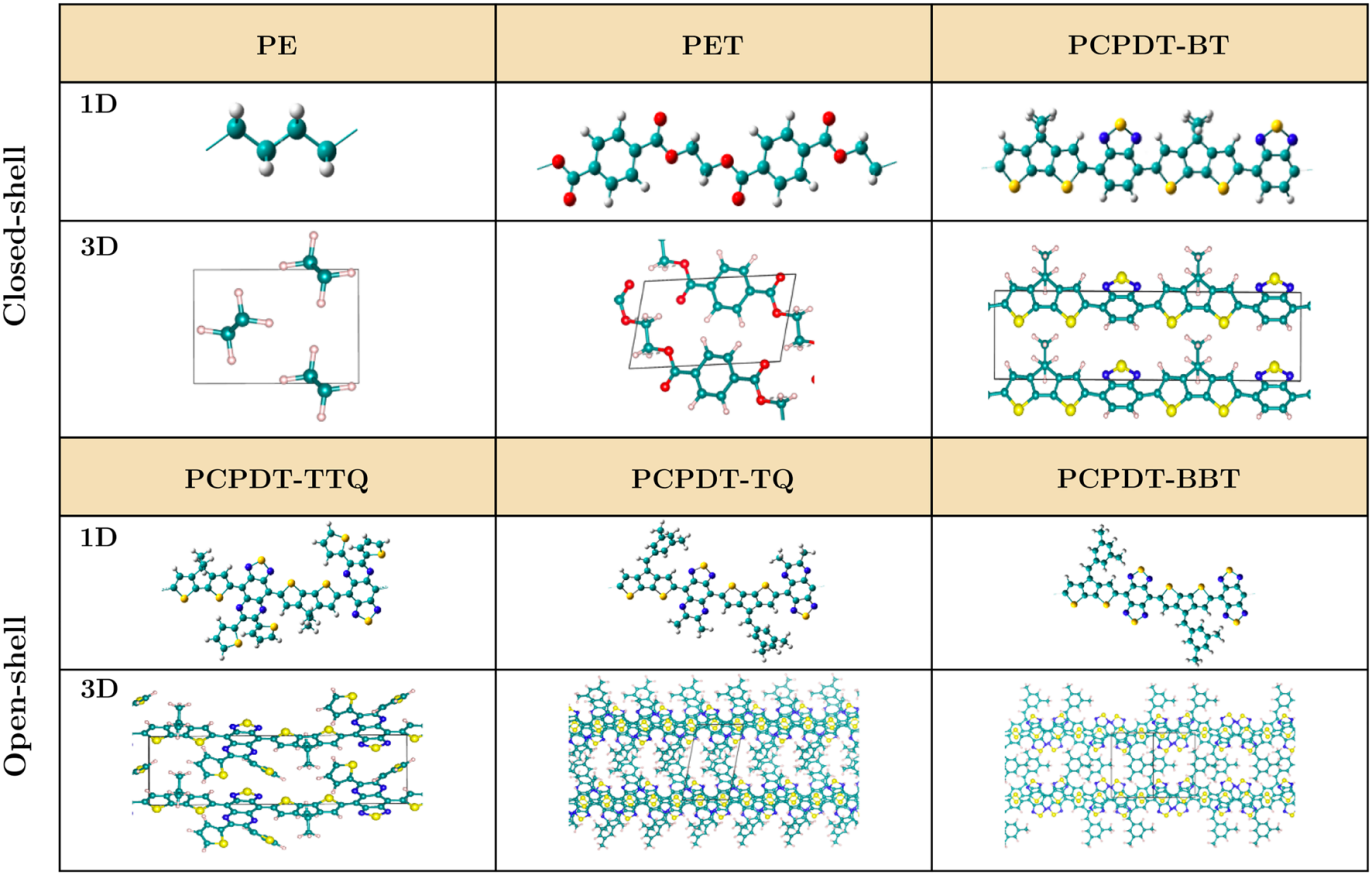
Optimized geometry of 1D and 3D models of organic polymers of PE, PET, PCPDT-BT, PCPDT-TTQ, PCPDT-TQ and PCPDT-BBT computed using DFT. The C, H, O, S, and N atoms are shown in cyan, white, red, yellow, and blue colors, respectively.

Here, we investigate the electronic structure and optical response of various cyclopentadithiophene-based D–A organic polymers using DFT and BSE methods. Specifically, we concentrate on PCPDT-BT (poly(4-(4H-cyclopenta[2,1-b:3,4-b′]dithiophen-2-yl)-4,7-(2,1,3-benzothiadiazole)), PCPDT-TTQ (poly(4-(4H-cyclopenta[2,1-b:3,4-b′]dithiophen-2-yl)-6,7-di(thiophen-2-yl)[1,2,5]thiadiazolo[3,4-g]quinoxaline)), PCPDT-TQ (poly(4-(4-benzylidene)-4H-cyclopenta[2,1-b:3,4-b′]dithiophen-2-yl)-[1,2,5]-thiadiazolo[3,4-g]quinoxaline) and PCPDT-BBT (poly(4-(4-benzylidene)-4H-cyclopenta[2,1-b:3,4-b′]dithiophen-2-yl)-benzo[1,2-c;4,5-c′]bis[1,2,5]thiadiazole), which possesses acceptor units such as benzothiadiazole, thiadiazoloquinoxaline, benzobisthiadiazole and display open/closed-shell character. These polymers find applications in photovoltaics, PCPDT-BT, and its analogs are one of the well-known D–A systems^29–32^. Further, PCPDT-TTQ has been identified as a promising material for supercapacitors due to its stable and tunable redox activity^33^. PCPDT-TQ is known for its high-spin ground state with a narrow bandgap and weak exchange coupling between unpaired electrons^14^. It is to be noted that TTQ and TQ differ by an additional dithiophene which significantly changes the structural properties. All the BBT-based polymer possesses diradical character and reduced singlet-triplet gap^34–36^ with localized spins at the terminals of the polymer backbone^37^. Additionally, we investigate the well-known closed-shell polymers, polyethylene (PE) and polyethylene terephthalate (PET), as these provide good experimental data for the benchmark. PE and PET are widely available and commercially accessible polymers with distinct structural, electrical, thermal, and optical properties^38–41^. We include these polymers in the present work to allow for comparative analysis, providing valuable insights into the influence of extended/limited $\pi$-electron arrangement and aromaticity on the electronic structure properties among PE, PET, and D–A polymers. In Fig. 1, we show the optimized geometries of one-dimensional (1D) and three-dimensional (3D) models of PE, PET, PCPDT-BT, PCPDT-TTQ, PCPDT-TQ, and PCPDT-BBT polymers computed using DFT by applying periodic boundary condition.

## Results

The 1D model provides an excellent approximation for analyzing a single, isolated polymer chain, while 3D models allow us to incorporate weak intermolecular interactions by including van der Waals contributions through dispersion corrections, such as Grimme’s D3 approach^42^. Each unit cell contains two monomer units, except for 3D PET, which contains only one monomer. The 3D D–A polymers and PET exhibit the triclinic phase, while PE shows orthorhombic. Structural parameters of the crystalline phase of different polymers, including known experimental values, are provided in Table S1 in the Supplementary Information. Good agreement between the computed and experimental results is observed for PE and PET. The low density (1.4-1.6 g/cm$^3$) of the D–A polymers compared to conventional inorganic semiconductors (Si = 2.33 g/cm$^3$ and GaAs = 5.32 g/cm$^3$)^43^ suggests that these polymers have potential applications in lightweight electronic devices.

**Figure 2:**
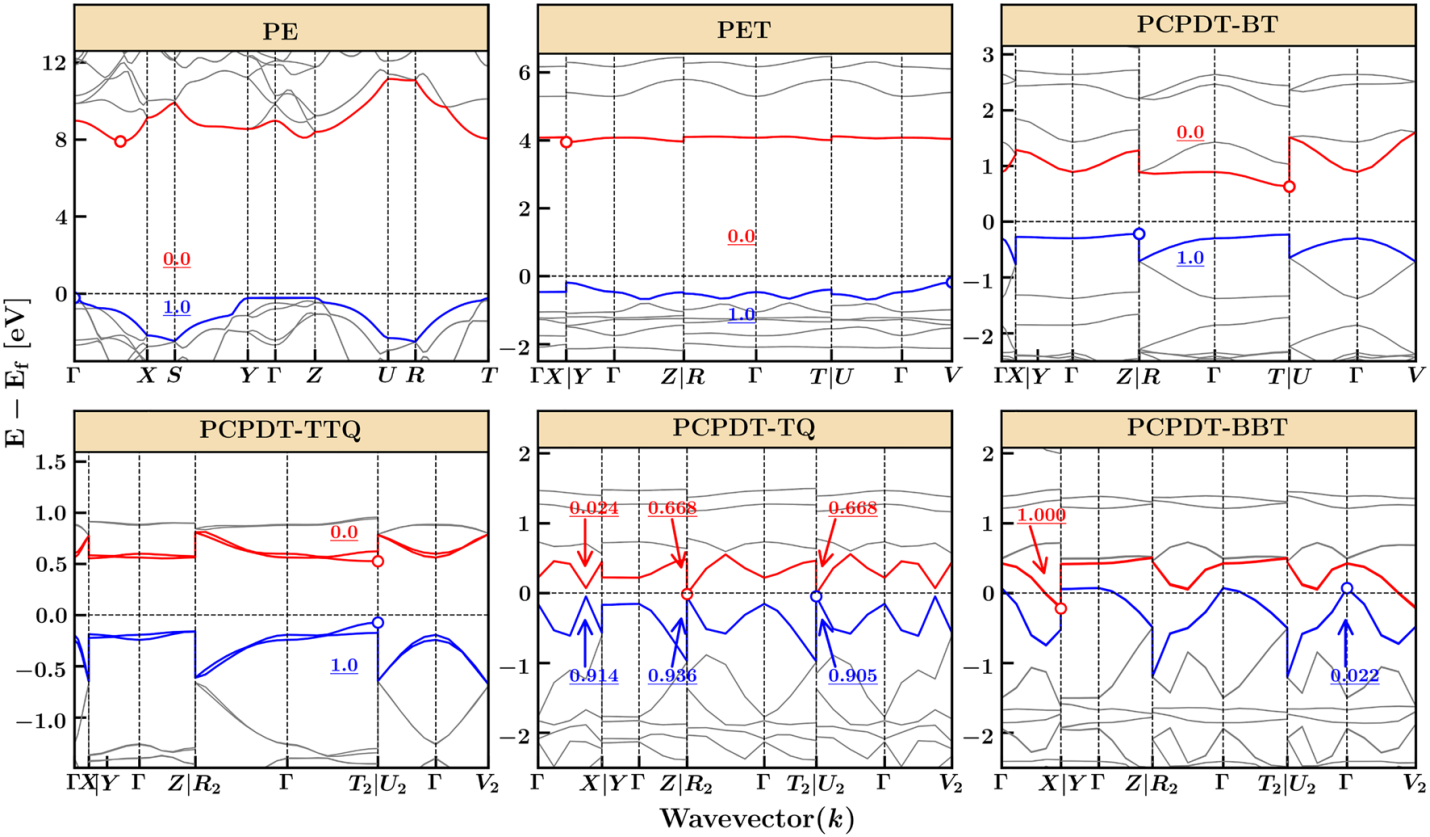
Band structure for PCPDT-BT, PCPDT-TTQ, PCPDT-TQ, and PCPDT-BBT along the high symmetry points computed using HSE06 method. VBM-1, VBM, CBM, and CBM+1 states are highlighted in colors. The electron occupancies along selected wave vectors are also shown. VBM and CBM are marked with “o”.

Unlike small open-shell organic molecules, conjugated organic polymers with a high spin ground state are rarely reported in their neutral form^10,14,44^. Due to the $\pi$-conjugated backbone, identifying the localized spin states is often challenging. While magnetic measurements such as superconducting quantum interference device (SQUID) and electron paramagnetic resonance (EPR) spectra can help identify high spin states^10,14^, the nature of spin-localization in the polymer remains unclear. To gain a better understanding of the electronic arrangement, multi-radical character, valance band (VB), and conduction band (CB) splitting, we have used a hybrid Heyd-Scuseria-Ernzerhof (HSE06) DFT functional to evaluate the band structure of these polymers. Figure 2 displays the band structure highlighting VB, VB-1, CB, and CB+1 states, with circles representing the valence band maximum (VBM) and the conduction band minimum (CBM). We observe degenerate energy levels between VB & VB-1 and CB & CB+1 with an indirect bandgap for PE, PET, PCPDT-BT, and PCPDT-TQ, non-degenerate levels for PCPDT-TTQ, and nearly degenerate levels for PCPDT-BBT. Further, the CBM of the PCPDT-TTQ and PCPDT-TQ polymers touches the Fermi level, and the CBM of PCPDT-BBT is below the VBM, exhibiting semi-metallic behavior. To understand the discrepancies in these polymers’ electronic arrangements, we evaluated the electron occupancies along various ${{\textbf {k}}}$-points shown in Fig. 2 and Table S2 and S3. We observe a full electron occupancy (1.0*e*) in VB and a zero-electron occupancy (0.0*e*) in CB in PE, PET, and PCPDT-BT, revealing their closed-shell character with a non-zero electronic bandgap. However, PCPDT-TQ shows a CB closer to the Fermi level with non-zero electron occupancy along various ${{\textbf {k}}}$-points, indicating open-shell character. Unlike other polymers, PCPDT-BBT exhibits a full occupancy with 1.0*e* in CBM, followed by 0.022e in VBM. The strong quinoidal nature of TQ, and BBT acceptors is crucial in lowering the CB and stabilizing unpaired electrons in the long-chain limit. These results align with previous experiments/computations demonstrating the open-shell character of PCPDT-TQ, and PCPDT-BBT polymers^14,37^.

**Figure 3:**
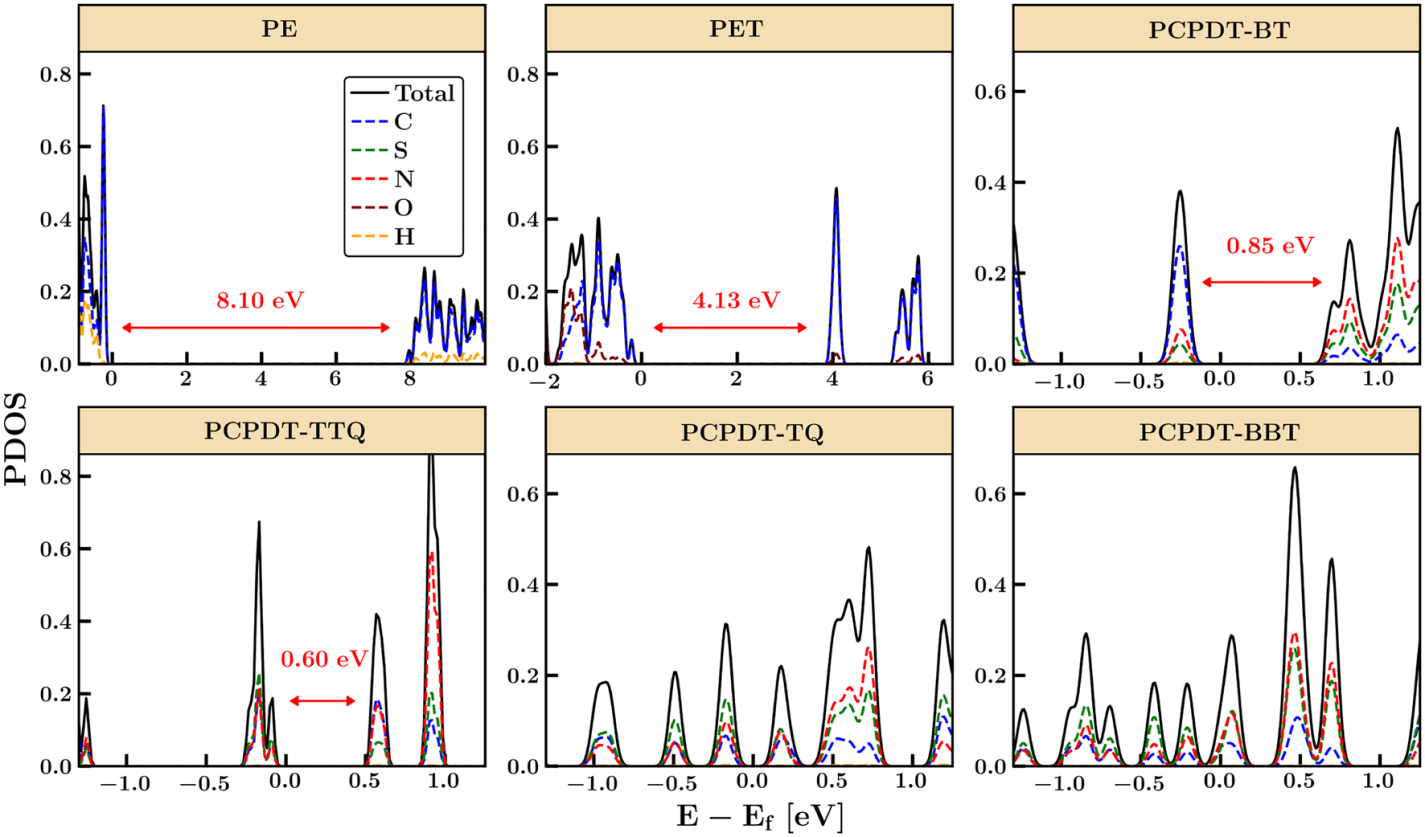
Projected density of states (PDOS) of organic polymers of PE, PET, PCPDT-BT, PCPDT-TTQ, PCPDT-TQ, and PCPDT-BBT computed using HSE06 method. Total DOS and the relative contributions of the individual atoms of the C, S, N, O and H atoms are shown. The computed electronic bandgaps obtained from band structure for PE, PET, and PCPDT-BT are also shown.

To gain insight into the electronic properties and charge transport behavior of various polymers, we employed the HSE06 method to analyze their partial (s, P$_x$, P$_y$, and P$_z$ orbitals) and projected (contributions from C, N, S, O, and H atoms) density of states (DOS) contributions. To compare the results across all polymers, we presented the normalized PDOS and total DOS in Fig. 3, with the partial DOS shown in Figures S1, S2, and S3. Our analysis revealed that the VB and CB edges primarily comprise sp$^2$-hybridized $\pi$ bonding and $\pi ^*$ antibonding orbitals, respectively, except PE, which is determined by sp$^3$-hybridized $\sigma$ bonding and $\sigma ^*$ antibonding orbitals from the P$_x$-orbital. In some polymers, such as PET, conjugation with a lateral overlap of P$_z$-orbitals introduces localized states that lower the band gap. Other polymers, such as PCPDT-BT and PCPDT-TTQ, display extensive conjugation with high contributions from P$_z$-orbitals in both the VB and CB edges. Adding the benzyl group to PCPDT in PCPDT-TQ and PCPDT-BBT through sp$^2$-hybridized carbon leads to a higher contribution from P$_y$-orbital in both VB and CB edges. We further examined the local DOS to identify the contributions from donor, acceptor, and substitution units in the D–A polymers, with results shown in Figure S4 in SI. Our computations demonstrate that the localization/delocalization and electronic excitations are strongly affected by the donor and acceptor units and the $\pi$-electrons. We also observed that all conjugated polymers’ VB and CB edges have a relatively substantial contribution from C, N, O, and S atoms in different proportions, indicating that electron transition most likely involves a strong correlation between the electrons along the polymer backbone.

**Table 1:** Electronic bandgaps computed using DFT and Macroscopic static dielectric constant ($\varepsilon$) computed using density functional perturbation theory (DFPT) and BSE for PE, PET, PCPDT-BT, PCPDT-TTQ, PCPDT-TQ, and PCPDT-BBT.

	Electronic Bandgap (eV)				Optical gap (eV)	Static dielectric constant ($\varepsilon$)		
	(Expt.)$^i$	Octamer (y$_0$)	1D	3D (Occ)	(Expt)$^{ii}$	BSE	DFPT	(Expt.)$^{iii}$
		B3LYP	B3LYP	HSE06				
PE	–	5.28 (0.0)	9.89	8.10	8.8	1.84	2.87	2.25
PET	–	5.11 (0.0)	5.28	4.13	4.0	1.56	2.36	$\sim$ 3.5
PCPDT-BT	1.81	1.44 (0.0)	1.78	0.85 (0.0)	1.44	8.82	12.40	3.6
PCPDT-TTQ	0.80	0.80 (0.98)	0.88	0.60 (0.0)	$\sim$ 0.46	5.49	8.99	–
PCPDT-TQ	0.6	0.77 (0.91)	0.36	− (0.67)	0.30	10.18	14.62	–
PCPDT-BBT	–	0.88 (1.00)	1.18	− (1.00)	–	13.73	32.04	–

For octamers, the computations are carried out using B3LYP at 6-31G(d,p) level. The occ in the parenthesis represents the probability amplitude of finding an electron which is the electron occupancy in the conduction band minimum level. ${^i}$, $^{ii}$, and $^{iii}$ represent an experimental electrochemical gap in the solution phase, absorption onsets from crystalline/thin films, and known experimental dielectric constant, respectively^14,33,37,45–50^.

Table 1 summarizes electronic bandgap values computed using B3LYP (Becke, three-parameter exchange functional; Lee, Yang, and Parr) and HSE06 functionals for 1D and 3D models of polymers, along with previous computational and experimental data. PE, a long hydrocarbon chain, is an insulator with a large bandgap of 8.10 eV, which is in close agreement with the measured optical bandgap of 8.8 eV^16,46^. On the other hand, PET with aromatic rings and C=O groups leads to delocalization of $\pi$-orbitals yielding a lower bandgap compared to PE. HSE06 predicts the electronic bandgap of PET to be 4.13 eV, which agrees with the experimental optical gap of 4.0 eV^47^. The electronic bandgap of 1D PE and PET computed using B3LYP is at the upper bound due to the absence of intermolecular interactions. For 1D PCPDT-BT and PCPDT-TTQ, B3LYP yields electronic bandgap values of 1.78 and 0.88 eV, respectively, which agree well with measured values of 1.81 and 0.80 eV^33,45^. However, B3LYP underestimates the electronic gap of PCPDT-TQ by 0.2 eV. Broken-symmetry DFT approach for linear single molecular D–A octamers predicts electronic bandgap values that are in good agreement with experiments^14,37^. The bandgap of 3D and octamer models of PCPDT-BT is underestimated compared to the experimental electronic and optical gap. However, for PCPDT-TTQ, the fundamental gap of 0.6 eV agrees well with the measured optical gap. In contrast, for PCPDT-TQ, and PCPDT-BBT, the CBM aligns along the Fermi level, resulting in zero bandgap with Dirac points, similar to Dirac materials, due to crystalline order^51–53^. The G$_0$W$_0$ quasiparticle energy gaps provide more reliable results compared to the HSE06 method. To better understand the discrepancies, we also calculated the quasiparticle energy band gap using the G$_0$W$_0$ approach. However, due to the utilization of a coarse *k*-point grid in our G$_0$W$_0$ calculation, we do not have access to the quasiparticle values required for the estimation of the exact band gap. Nonetheless, we have computed the band gap at the $\Gamma$-point and have included a comparison of the G$_0$W$_0$, HSE06, and PBE methods in Table S4 of the SI. As shown in the table, we observe an expected increase in the bandgap values from PBE, HSE06 to G$_0$W$_0$. We anticipate that a similar increase in the bandgap values can be expected at other *k* -points as well which may result in finite gap values for PCPDT-BBT and PCPDT-TQ which are otherwise nonexistent at the HSE06 level of calculations.

**Figure 4:**
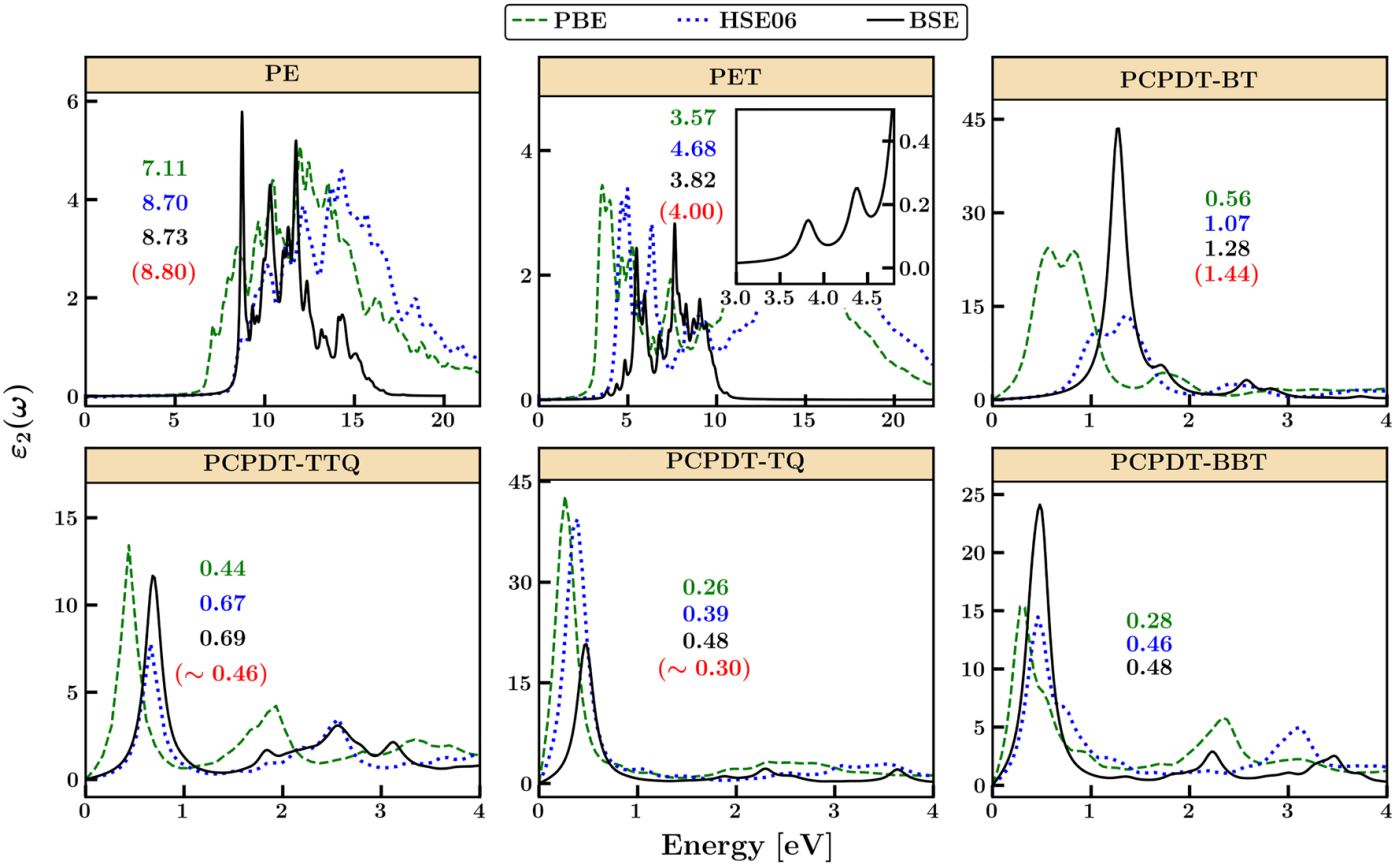
The imaginary part of the dielectric function ($\varepsilon _2(\omega )$) for the 3D organic polymers of PE, PET, PCPDT-BT, PCPDT-TTQ, PCPDT-TQ, and PCPDT-BBT computed using PBE, HSE06 and BSE method. The first exciton peak and the experimental optical gap (in parenthesis)^14,33,45–47^ obtained from the absorption onset are also shown. Inset in PET and PCPDT-TTQ shows the first exciton peak computed at BSE and HSE06, respectively.

It should be noted that in organic semiconductors, the electronic bandgaps determined from band structures are insufficient for describing the optical gap, unlike in inorganic semiconductors. This discrepancy is due to the weak electronic coupling in organic semiconductors, resulting in the formation of excitons rather than charge carriers upon photoexcitation, with an exciton binding energy of a few hundred meV. To gain insight into photoexcitation, we used PBE (Perdew-Burke-Ernzerhof) and HSE06 functionals to compute optical spectra and solve BSE on top of G$_0$W$_0$ approximation to account for excitonic effects^54–56^. The optical spectra, as shown in Fig. 4, exhibit strong absorption in the ultraviolet, visible, and near-infrared regions. While PE and PET exhibit a broad spectrum, sharp and intense peaks are observed in D–A polymers. As anticipated, the spectra obtained from PBE are red-shifted compared to those obtained from HSE06. The first peak from HSE06 and BSE aligns well with the measured optical bandgap from the absorption onset.

Subsequently, we employed the BSE and density functional perturbation theory (DFPT) to compute the static dielectric constant, as shown in Table 1 and S6. Our computed values for PE and PET are in close agreement with experimental results. However, for donor-acceptor (D–A) polymers, we observe a high dielectric constant owing to their small band gap resulting in a tiny energy denominator (see Methods section). The computed dielectric constants for PCPDT-BT, PCPDT-TTQ, PCPDT-TQ, and PCPDT-BBT, exhibiting finite or zero band gaps, are 12.40, 8.99, 14.62, and 32.04, respectively. Upon detailed analysis of the dielectric constant tensor (as shown in Table 1 and Table S5), it is evident that the primary contribution arises from the tensor component aligned with the polymer backbone ($\epsilon _{xx}$), while the components perpendicular to the backbone axis ($\epsilon _{yy}$ and $\epsilon _{zz}$) remain relatively low. We also observe a direct correlation between the dielectric constant and the electron occupancy (as indicated by Occ in Table 1) in the conduction band (CB), indicating that the presence of localized spins in open-shell systems enhances the dielectric properties of these materials. Recent experimental work on PCPDT-TTQ has demonstrated its suitability as an *n*-type material for its applications as Faradaic supercapacitors (as referenced in^33^). Consequently, the significant dielectric enhancement due to localized spins highlights the versatility of open-shell donor-acceptor (D–A) polymers, positioning them for a wide range of applications, including organic electronics and the production of dielectric films.

## Discussion

An improved understanding of organic polymers with open-shell character is necessary to realize their full potential in photonics, spintronics, and quantum computing applications. Here, using DFT and high-level *ab initio* methods, such as the many-body perturbation-based GW approximation and BSE, we elucidate the electronic structure and optical properties of CPDT-based D–A organic polymers. We find that the electronic and optical properties are significantly influenced by the donor and the acceptor units and $\pi$-spacers. In particular, using DFT and BSE based on the G$_0$W$_0$ approach, we have analyzed the band structure and optical spectra. Our results indicate a redistribution of electrons along different ${{\textbf {k}}}$-points due to the lowering of CB for open-shell polymers. The electronic bandgap computed for PCPDT-BT and PCPDT-TTQ 1D polymers and PCPDT-TTQ, PCPDT-TQ, and PCPDT-BBT octamers agrees well with experimental measurements. We also observed excitonic peaks resulting from transitions to unoccupied bands, complementing the measured optical bandgaps of crystalline/thin films. D–A polymers have the potential for near-infrared active semiconducting materials and spintronics applications due to the narrow optical bandgap of $\sim$0.5 eV and inherent open-shell characteristics. Our computational model and methodology show potential in determining the optoelectronic properties of organic polymers. However, a key challenge for future studies is to expand the model to include disorders, defects, alkyl side chains, temperature, and pressure and to model semi-crystalline or amorphous phases using classical and *ab initio* molecular dynamics simulations. By doing so, we can establish the groundwork for improved modeling of D–A semiconducting materials, extending the range of computational methods for systematically screening and exploring new materials for optoelectronics and spintronics.

## Methods

Geometry optimizations of all the three-dimensional (3D) model polymers are carried out using density functional theory (DFT) by imposing periodic boundary conditions as implemented in the VASP code^57,58^. The study commenced with benchmark calculations, predicated on chemical intuition, utilizing diverse configurations in 3D crystalline phases, such as stacked and slipped, as the initial geometries. Optimization of the lattice parameters and atomic positions was conducted for each configuration. The minimum energy configuration was subsequently identified for comprehensive analysis of the electronic structure and optical properties. The projector augmented wave (PAW) pseudopotential is used with Perdew-Burke-Ernzerhof (PBE) functional^59^. The van der Waals interactions are included using the DFT-D3 method of Grimme et al^42^. A recommended cutoff energy of 500 eV is used to generate a plane-wave basis set^60,61^. A Monkhorst-Pack *k*-point mesh ($\Gamma$-centered) is employed for the Brillouin zone integration. To fix the density of the *k*-grid, we used VASPKIT which uses $k=V_{cell}/N_{kpt}$ where *k* is the precession (distance between two adjacent *k*-points), $V_{cell}$ volume of the cell, and $N_{kpt}$ is the number of *k* points. Given the significantly large systems considered in the present work, we chose $k=0.04$, which strikes a good balance between accuracy and computational cost. The self-consistent field energies are converged with a tolerance of 10$^{-5}$, and forces on each atom are less than 0.02 eV/Å. The electrons in open-shell D–A polymers are strongly correlated and involve a change in spin states. We include the spin-orbit coupling (SOC) in our calculations through the noncollinear spin-polarized method as implemented in VASP where the spin-polarization of the electronic structure is represented by a three-dimensional vector at each atom in the system. It further affects the rate of electron transfer between the donor and acceptor by altering the energy levels of electrons involved in the transfer. The relaxed 3D geometries are further used to compute the crystalline electronic bandgap using Heyd-Scuseria-Ernzerhof (HSE06) functional^62^ with a screening parameter set to 0.2 bohr$^{-1}$. To evaluate the band structure, VASPKIT^63^ is used to generate the k-points covering high-symmetry points in the Brillouin zone. Many-body effects are taken into account by computing the quasiparticle (QP) energies using GW calculations (where G is the Green’s function, and W stands for screened Coulomb interaction) with a standard one-shot G$_0$W$_0$ approach^64–66^, using a starting wavefunction taken from PBE method with few numbers of ${{\textbf {k}}}$-points. Table S6 in SI shows the number of k-points and empty levels considered for various polymers. To determine the dielectric function and optical spectrum, incorporating electron-hole interactions, we solve the Bethe–Salpeter equation^54,55,67^ on top of G$_0$W$_0$ calculation within the Tamm-Dancoff approximation^68^. This approach effectively characterizes the polymer’s optical response to electromagnetic radiation and effectively locates the exciton peaks. We calculate the real ($\varepsilon _1$) and imaginary ($\varepsilon _2$) components of the dielectric matrix, representing the dielectric constant and dielectric loss, respectively. The imaginary part of the dielectric function is derived from the momentum matrix elements between occupied and unoccupied wave functions.

$$\begin{aligned} \varepsilon _{2}({\textbf{q}},\omega ) = \frac{4 \pi ^2 e^2}{\Omega }\lim _{{{{\textbf {q}}}}\rightarrow 0} \frac{1}{\mid {{{\textbf {q}}}}\mid ^2} \sum _{c, v,{{{\textbf {k}}}}} 2w_{{{{\textbf {k}}}}} \delta ( {\epsilon _{c{{{\textbf {k}}}}+{{{\textbf {q}}}}}-\epsilon _{v{{{\textbf {k}}}}}}-\omega ) \mid <u_{c{{{\textbf {k}}}}+{{{\textbf {q}}}}}\mid u_{v{{{\textbf {k}}}}}>\mid ^2. \end{aligned}$$

While the real part can be evaluated from $\varepsilon _2$ using the Kramers–Kronig transformation

$$\begin{aligned} \varepsilon _{1}(\omega ) = 1+ \frac{2}{\pi }P \int _{0}^{\infty }\frac{\varepsilon _{2}(\omega ')\omega '}{\omega '^2 - \omega ^2} d\omega ' \end{aligned}$$

Where $\epsilon _{v{{{\textbf {k}}}}}$ and $\epsilon _{c{{{\textbf {k}}}}+{{\textbf {q}}}}$ are energies of VB and CB states. $\Omega$, ${{{\textbf {q}}}}$, $w_{{{{\textbf {k}}}}}$, and $u_{c{{{\textbf {k}}}}}$ are cell volume, Bloch vector of the incident wave, k-point weights, and cell periodic part of the wave function, respectively.

The first peak in the imaginary part of the dielectric spectrum represents the optical bandgap^26,54^. To obtain the dielectric spectra without any local effects, the frequency-dependent dielectric matrix is evaluated from the electronic ground state (PBE and HSE06) using independent particle approximation^56^.

Further, the macroscopic dielectric constant tensors are evaluated using density functional perturbation theory (DFPT) using PBE functional^69,70^. The random phase approximation accounts for the local field effects corresponding to the cell’s periodic part of the potential. The Dyson equation shows that the dielectric constant is related to polarizability ($\chi$), $\varepsilon = 1+\nu \chi$, $\nu$ being Coulomb kernel. Typically, the static dielectric matrix can be obtained from the first-order response of the wave function to an externally applied field with the inner product of the change in wave function and polarization vector ($\vec {\beta }_{v{{{\textbf {k}}}}}$). The polarizability is inversely proportional to the sum-over the states of difference in energies between VB and CB states as well as the polarization in the cell periodic part of the wavefunction ($u_{c{{{\textbf {k}}}}}$).

$$\begin{aligned} \chi ^0_{0,0}({{{\textbf {{q}}}}},0) = -\frac{2\mid {{{\textbf {q}}}} \mid ^2}{\Omega } \sum _{c,v,{{{\textbf {k}}}}} 2w_{{{{\textbf {k}}}}} \frac{<{{\textbf {{q}}}}\vec {\beta }_{v{{{\textbf {k}}}}}\mid u_{c{{{\textbf {k}}}}}> <u_{c{{{\textbf {k}}}}}\mid {{\textbf {{q}}}}\vec {\beta }_{v{{{\textbf {k}}}}}>}{\epsilon _{c{{{\textbf {k}}}}+{{{\textbf {q}}}}}-\epsilon _{v{{{\textbf {k}}}}}}. \end{aligned}$$

Thus, high polarizability and the low band gap enhance the dielectric constant. We would like to draw attention to an important consideration regarding the calculation of the dielectric constant for PCPDT-TTQ. To mitigate potential numerical instabilities, it is advisable to use a denser *k*-grid with $k < 0.04$. This increased grid density ensures more accurate and reliable calculations of the dielectric constant, avoiding any inaccuracies that may arise from inadequate sampling. However, employing a denser *k*-grid can significantly increase the computational costs for GW and BSE calculations. To address this issue, we employed a shifted $\Gamma$-centered or Monkhorst Pack *k*-grid for PCPDT-TTQ (see Supplementary Information, Table S6), effectively mitigating the aforementioned numerical instabilities associated with this system. A similar *k*-grid was also utilized for other systems to evaluate the dielectric constant at the DFPT level, yielding comparable results to those obtained with a $\Gamma$-centered grid (see Supplementary Information, Table S7). The observed differences in the dielectric constant values at the DFPT level across different *k*-grid choices can be attributed to the errors introduced by the choice of *k*-grid density.

For one-dimensional (1D) model polymers and octamers, the B3LYP^71^ method with 6-31g(d,p) basis set is employed using Gaussian 16 software^72^. A broken-symmetry wave function is used to characterize the open-shell character for octamers. The open-shell character can be quantitatively described using the multi-radical indexes (${\text{y}_i}, \;\,0 \le{\text{y}_i} \le1$)^73,74^. The diradical (y$_0$) index is obtained from the occupation number of the lowest unoccupied natural orbital (LUNO) with y${_0}=0$ and y${_0}=1$ referring to a pure close- and open-shell character, respectively.

We note that the calculations that we performed are computationally costly. The optical spectra using BSE based on the G$_0$W$_0$ method for D–A polymers are carried out in the in-house High-Performance Computing Collaboratory (HPC$^2$) at Mississippi State University. We used 30–40 nodes for each calculation with dual 20-core Xeon Gold 6148 processors and approximately 6 to 7-terabyte memory. We find an increase in memory requirement with ${{\textbf {k}}}$-points. The optical spectra and band structure calculations using HSE06 are carried out using Extreme Science and Engineering Discovery Environment (XSEDE)^75^ with 1500+ cores and 5 to 6 terabyte memory.

### Supplementary Information

Supplementary Information.

## Data Availability

All the relevant data of this study are available in the Supplementary Information. This includes Supplementary Data structural parameters of a one-dimensional and three-dimensional model of organic polymers, electronic eigenstates, and electron occupancies in valence and conduction band along various ${{\textbf {k}}}$-points in PCPDT-TTQ, PCPDT-TQ and PCPDT-BBT polymers computed using HSE06 method, the partial and local density of states, static dielectric tensors with $\Gamma$- centered and the Monkhorst-Pack schemes, band gaps at $\Gamma$-point, number of ${{\textbf {k}}}$-points and empty bands used in G$_0$W$_0$ and DFPT and coordinates of polymers.

## References

[1] Sekine C, Tsubata Y, Yamada T, Kitano M, Doi S (2014). Recent progress of high performance polymer OLED and OPV materials for organic printed electronics. Sci. Technol. Adv. Mater..

[2] Zhang, B., Chen, Y., Neoh, K.-G., Kang, E.-T.: *CHAPTER 1 Organic Electronic Memory Devices*, 1–53. (The Royal Society of Chemistry, 2016). 10.1039/9781782622505-00001

[3] Su Y-W, Lan S-C, Wei K-H (2012). Organic photovoltaics. Mater. Today.

[4] Nakano M, Champagne B (2015). Theoretical design of open-shell singlet molecular systems for nonlinear optics. J. Phys. Chem. Lett..

[5] Roberts ME, Wheeler DR, McKenzie BB, Bunker BC (2009). High specific capacitance conducting polymer supercapacitor electrodes based on poly(tris(thiophenylphenyl)amine). J. Mater. Chem..

[6] Rahman MA, Kumar P, Park D-S, Shim Y-B (2008). Electrochemical sensors based on organic conjugated polymers. Sensors.

[7] Lin Y, Li Y, Zhan X (2012). Small molecule semiconductors for high-efficiency organic photovoltaics. Chem. Soc. Rev..

[8] Bujak P, Kulszewicz-Bajer I, Zagorska M, Maurel V, Wielgus I, Pron A (2013). Polymers for electronics and spintronics. Chem. Soc. Rev..

[9] Huang Y, Egap E (2018). Open-shell organic semiconductors: An emerging class of materials with novel properties. Poly. J..

[10] Chen Z, Li W, Sabuj MA, Li Y, Zhu W, Zeng M, Sarap CS, Huda MM, Qiao X, Peng X, Ma D, Ma Y, Rai N, Huang F (2021). Evolution of the electronic structure in open-shell donor–acceptor organic semiconductors. Nat. Commun..

[11] Lombardi F, Lodi A, Ma J, Liu J, Slota M, Narita A, Myers WK, Müllen K, Feng X, Bogani L (2019). Quantum units from the topological engineering of molecular graphenoids. Science.

[12] Müllen K, Pisula W (2015). Donor-acceptor polymers. J. Am. Chem. Soc..

[13] Zeng Z, Ishida M, Zafra JL, Zhu X, Sung YM, Bao N, Webster RD, Lee BS, Li R-W, Zeng W, Li Y, Chi C, Navarrete JTL, Ding J, Casado J, Kim D, Wu J (2013). Pushing extended p-Quinodimethanes to the limit: Stable tetracyano-oligo(N-annulated perylene)quinodimethanes with tunable ground states. J. Am. Chem. Soc..

[14] London AE (2019). A high-spin ground-state donor–acceptor conjugated polymer. Sci. Adv..

[15] Mahesh K, Karpagam S, Pandian K (2019). How to design donor–acceptor based heterocyclic conjugated polymers for applications from organic electronics to sensors. Top. Curr. Chem. (Z).

[16] Chen L, Batra R, Ranganathan R, Sotzing G, Cao Y, Ramprasad R (2018). Electronic structure of polymer dielectrics: The role of chemical and morphological complexity. Chem. Mater..

[17] Turan HT, Kucur O, Kahraman B, Salman S, Aviyente V (2018). Design of donor-acceptor copolymers for organic photovoltaic materials: A computational study. Phys. Chem. Chem. Phys..

[18] Vukmirović N, Wang L-W (2009). Electronic structure of disordered conjugated polymers: Polythiophenes. J. Phys. Chem. B.

[19] Wong BM, Cordaro JG (2011). Electronic properties of vinylene-linked heterocyclic conducting polymers: Predictive design and rational guidance from dft calculations. J. Phys. Chem. C.

[20] Alesadi A, Fatima F, Xia W, Kilin D (2021). First-principles study on the electronic properties of PDPP-based conjugated polymer via density functional theory. J. Phys. Chem. B.

[21] Ferretti A, Mallia G, Martin-Samos L, Bussi G, Ruini A, Montanari B, Harrison NM (2012). Ab initio complex band structure of conjugated polymers: Effects of hybrid density functional theory and $${GW}$$ schemes. Phys. Rev. B.

[22] Pandey L, Risko C, Norton JE, Brédas J-L (2012). Donor-acceptor copolymers of relevance for organic photovoltaics: A theoretical investigation of the impact of chemical structure modifications on the electronic and optical properties. Macromolecules.

[23] Zhang Z, Wang J (2012). Structures and properties of conjugated donor–acceptor copolymers for solar cell applications. J. Mater. Chem..

[24] Hachmann J, Olivares-Amaya R, Atahan-Evrenk S, Amador-Bedolla C, Sánchez-Carrera RS, Gold-Parker A, Vogt L, Brockway AM, Aspuru-Guzik A (2011). The Harvard clean energy project: Large-scale computational screening and design of organic photovoltaics on the world community grid. J. Phys. Chem. Lett..

[25] Kanal IY, Owens SG, Bechtel JS, Hutchison GR (2013). Efficient computational screening of organic polymer photovoltaics. J. Phys. Chem. Lett..

[26] Kronik L, Neaton JB (2016). Excited-state properties of molecular solids from first principles. Ann. Rev. Phys. Chem..

[27] Manna AK, Refaely-Abramson S, Reilly AM, Tkatchenko A, Neaton JB, Kronik L (2018). Quantitative prediction of optical absorption in molecular solids from an optimally tuned screened range-separated hybrid functional. J. Chem. Theory Comput..

[28] Prentice JCA, Mostofi AA (2021). Accurate and efficient computation of optical absorption spectra of molecular crystals: The case of the polymorphs of roy. J. Chem. Theory Comput..

[29] Zhou Y, Eck M, Veit C, Zimmermann B, Rauscher F, Niyamakom P, Yilmaz S, Dumsch I, Allard S, Scherf U, Krüger M (2011). Efficiency enhancement for bulk-heterojunction hybrid solar cells based on acid treated CdSe quantum dots and low bandgap polymer PCPDTBT. Sol. Energy Mat. Sol. C.

[30] Mühlbacher D, Scharber M, Morana M, Zhu Z, Waller D, Gaudiana R, Brabec C (2006). High photovoltaic performance of a low-bandgap polymer. Adv. Mat..

[31] Kowalski S, Allard S, Scherf U (2012). Synthesis of poly(4,4-dialkyl-cyclopenta[2,1-b:3,4-b’]dithiophene-alt-2,1,3-benzothiadiazole) (PCPDTBT) in a direct arylation scheme. ACS Macro Lett..

[32] Peet J, Kim JY, Coates NE, Ma WL, Moses D, Heeger AJ, Bazan GC (2007). Efficiency enhancement in low-bandgap polymer solar cells by processing with alkane dithiols. Nat. Mater..

[33] Wang K, Huang L, Eedugurala N, Zhang S, Sabuj MA, Rai N, Gu X, Azoulay JD, Ng TN (2019). Wide potential window supercapacitors using open-shell donor–acceptor conjugated polymers with stable n-doped states. Adv. Energy Mater..

[34] Qian G, Dai B, Luo M, Yu D, Zhan J, Zhang Z, Ma D, Wang ZY (2008). Band gap tunable, donor-acceptor-donor charge-transfer heteroquinoid-based chromophores: Near infrared photoluminescence and electroluminescence. Chem. Mater..

[35] Thomas A, Bhanuprakash K, Prasad KMMK (2011). Near infrared absorbing benzobis(thiadiazole) derivatives: computational studies point to biradical nature of the ground states. J. Phys. Org. Chem..

[36] Tam TLD, Wu J (2015). Benzo[1,2-c;4,5-c’]bis[1,2,5]thiadiazole in organic optoelectronics: A mini-review. J. Mol. Eng. Mater..

[37] Sabuj MA, Huda MM, Sarap CS, Rai N (2021). Benzobisthiadiazole-based high-spin donor–acceptor conjugated polymers with localized spin distribution. Mater. Adv..

[38] Chen L, Huan TD, Ramprasad R (2017). Electronic structure of polyethylene: Role of chemical, morphological and interfacial complexity. Sci. Rep..

[39] Less KJ, Wilson EG (1973). Intrinsic photoconduction and photoemission in polyethylene. J. Phys. C: Solid State Phys..

[40] Neagu E, Pissis P, Apekis L (2000). Electrical conductivity effects in polyethylene terephthalate films. J. Appl. Phys..

[41] Jabarin SA (1982). Optical properties of thermally crystallized poly(ethylene terephthalate). Polym. Eng. Sci..

[42] Grimme S, Antony J, Ehrlich S, Krieg H (2010). A consistent and accurate ab initio parametrization of density functional dispersion correction (dft-d) for the 94 elements h-pu. J. Chem. Phys..

[43] Appendix G (2006). Properties of Si and GaAs.

[44] Tam TLD, Wu G, Chien SW, Lim SFV, Yang S-W, Xu J (2020). High spin pro-quinoid benzo[1,2-c;4,5-c]bisthiadiazole conjugated polymers for high-performance solution-processable polymer thermoelectrics. ACS Mater. Lett..

[45] Horie M, Kettle J, Yu C-Y, Majewski LA, Chang S-W, Kirkpatrick J, Tuladhar SM, Nelson J, Saunders BR, Turner ML (2012). Cyclopentadithiophene-benzothiadiazole oligomers and polymers; synthesis, characterisation, field-effect transistor and photovoltaic characteristics. J. Mater. Chem..

[46] Less KJ, Wilson EG (1973). Intrinsic photoconduction and photoemission in polyethylene. J. Phys. C: Solid State Phys..

[47] Breese M, Trautmann C, Vickridge IC, Wang Y (2000). Optical and electrical properties of some electron and proton irradiated polymers. Nucl. Instrum. Methods Phys. Res. B.

[48] Armin A, Stoltzfus DM, Donaghey JE, Clulow AJ, Nagiri RCR, Burn PL, Gentle IR, Meredith P (2017). Engineering dielectric constants in organic semiconductors. J. Mater. Chem. C.

[49] Sears, F. W., Zemansky, M. W. & Young, H. D. University Physics, 6th Edition. Addison-Wesley series, 929 (Addison-Wesley Publishing Company, 1994). https://books.google.co.in/books?id=XSjYygAACAAJ.

[50] Konieczna M, Markiewicz E, Jurga J (2010). Dielectric properties of polyethylene terephthalate/polyphenylene sulfide/barium titanate nanocomposite for application in electronic industry. Polym. Eng. Sci..

[51] Wehling TO, Black-Schaffer AM, Balatsky AV (2014). Dirac materials. Adv. Phys..

[52] Tawfik SA, Isayev O, Stampfl C, Shapter J, Winkler DA, Ford MJ (2019). Efficient prediction of structural and electronic properties of hybrid 2D materials using complementary DFT and machine learning approaches. Adv. Theory Simul..

[53] Liu Q, Zunger A (2017). Predicted realization of cubic Dirac fermion in quasi-one-dimensional transition-metal monochalcogenides. Phys. Rev. X.

[54] Albrecht S, Reining L, Del Sole R, Onida G (1998). Ab initio calculation of excitonic effects in the optical spectra of semiconductors. Phys. Rev. Lett..

[55] Rohlfing M, Louie SG (1998). Electron-hole excitations in semiconductors and insulators. Phys. Rev. Lett..

[56] Gajdoš M, Hummer K, Kresse G, Furthmüller J, Bechstedt F (2006). Linear optical properties in the projector-augmented wave methodology. Phys. Rev. B.

[57] Blochl PE (1994). Projector augmented-wave method. Phys. Rev. B.

[58] Kresse G, Joubert D (1999). From ultrasoft pseudopotentials to the projector augmented-wave method. Phys. Rev. B.

[59] Perdew JP, Burke K, Ernzerhof M (1996). Generalized gradient approximation made simple. Phys. Rev. Lett..

[60] Pilania G, Weis E, Walker EM, Gilbertson RD, Muenchausen RE, Simakov EI (2018). Computational screening of organic polymer dielectrics for novel accelerator technologies. Sci. Rep..

[61] Sharifzadeh S, Biller A, Kronik L, Neaton JB (2012). Quasiparticle and optical spectroscopy of the organic semiconductors pentacene and PTCDA from first principles. Phys. Rev. B.

[62] Heyd J, Scuseria GE (2004). Efficient hybrid density functional calculations in solids: Assessment of the Heyd–Scuseria–Ernzerhof screened coulomb hybrid functional. J. Chem. Phys..

[63] Wang V, Xu N, Liu J-C, Tang G, Geng W-T (2021). VASPKIT: A user-friendly interface facilitating high-throughput computing and analysis using VASP code. Comput. Phys. Commun..

[64] Hedin L (1965). New method for calculating the one-particle green’s function with application to the electron-gas problem. Phys. Rev..

[65] Strinati G, Mattausch HJ, Hanke W (1980). Dynamical correlation effects on the quasiparticle bloch states of a covalent crystal. Phys. Rev. Lett..

[66] Strinati G, Mattausch HJ, Hanke W (1982). Dynamical aspects of correlation corrections in a covalent crystal. Phys. Rev. B.

[67] Salpeter EE, Bethe HA (1951). A relativistic equation for bound-state problems. Phys. Rev..

[68] Sander T, Maggio E, Kresse G (2015). Beyond the Tamm–Dancoff approximation for extended systems using exact diagonalization. Phys. Rev. B.

[69] Gajdoš M, Hummer K, Kresse G, Furthmüller J, Bechstedt F (2006). Linear optical properties in the projector-augmented wave methodology. Phys. Rev. B.

[70] Nunes RW, Gonze X (2001). Berry-phase treatment of the homogeneous electric field perturbation in insulators. Phys. Rev. B.

[71] Becke AD (1993). Becke’s three parameter hybrid method using the LYP correlation functional. J. Chem. Phys..

[72] Frisch MJ, Trucks GW, Schlegel HB, Scuseria GE, Robb MA, Cheeseman JR, Scalmani G, Barone V, Petersson GA, Nakatsuji H, Li X, Caricato M, Marenich AV, Bloino J, Janesko BG, Gomperts R, Mennucci B, Hratchian HP, Ortiz JV, Izmaylov AF, Sonnenberg JL, Williams-Young D, Ding F, Lipparini F, Egidi F, Goings J, Peng B, Petrone A, Henderson T, Ranasinghe D, Zakrzewski VG, Gao J, Rega N, Zheng G, Liang W, Hada M, Ehara M, Toyota K, Fukuda R, Hasegawa J, Ishida M, Nakajima T, Honda Y, Kitao O, Nakai H, Vreven T, Throssell K, Montgomery JA, Peralta JE, Ogliaro F, Bearpark MJ, Heyd JJ, Brothers EN, Kudin KN, Staroverov VN, Keith TA, Kobayashi R, Normand J, Raghavachari K, Rendell AP, Burant JC, Iyengar SS, Tomasi J, Cossi M, Millam JM, Klene M, Adamo C, Cammi R, Ochterski JW, Martin RL, Morokuma K, Farkas O, Foresman JB, Fox DJ (2016). Gaussian 16 Revision B.01.

[73] Nakano M, Fukui H, Minami T, Yoneda K, Shigeta Y, Kishi R, Champagne B, Botek E, Kubo T, Ohta K, Kamada K (2011). (hyper)polarizability density analysis for open-shell molecular systems based on natural orbitals and occupation numbers. Theor. Chem. Acc..

[74] Gopalakrishna Y, Zeng T, Lu W, Wu X (2018). From open-shell singlet diradicaloids to polyradicaloids. J. Chem. Commun..

[75] Towns J, Cockerill T, Dahan M, Foster I, Gaither K, Grimshaw A, Hazlewood V, Lathrop S, Lifka D, Peterson GD, Roskies R, Scott JR, Wilkins-Diehr N (2014). XSEDE: Accelerating scientific discovery. Comput. Sci. Eng..

