# Supplementary material for: Electronic properties and optical spectra of donor–acceptor conjugated organic polymers
Source: Sci Rep. 2023 Dec 7;13:21587. doi: 10.1038/s41598-023-48468-9 (PMC10703904; doi:10.1038/s41598-023-48468-9)
Supplement: Supplementary file 1 — Supplementary Information. [file 41598_2023_48468_MOESM1_ESM.pdf]

Supporting Information: Electronic Properties and Optical Spectra of Donor-Acceptor Conjugated Organic Polymers

Chandra Shekar Sarap, Yashpal Singh, John Michael Lane, and Neeraj Rai*

Dave C. Swalm School of Chemical Engineering and Center for Advanced Vehicular Systems, Mississippi State University, Mississippi State, 39762, Mississippi, USA

E-mail: *neerajrai@che.msstate.edu

Table S1: Structural parameters of crystalline phase of 1D and 3D polymers of PE, PET, PCPDT-BT, PCPDT-TTQ, PCPDT-TQ, and PCPDT-BBT computed using DFT. Experimental lattice parameters and densities (ρ) for PE and PET are shown in parenthesis.[S1]

	1D	3D							
	a′	a	b	c	α	β	γ	ρ (g/cm^3)	k-points mesh
PE (C_4H_8)	5.13	2.55 (2.55)	6.75 (7.12)	4.67 (4.85)	90.0 (90.0)	90.0 (90.0)	90.0 (90.0)	1.16 (1.05)	10x4x5
PET ($C_{10}O_4H_8$)	21.77	9.68 (10.75)	5.72 (5.96)	3.92 (4.56)	74.88 (111.5)	77.25 (112.0)	74.50 (98.5)	1.60 (1.60)	2x3x4
PCPDT-BT ($C_{34}S_6N_4H_{20}$)	24.54	24.48	6.30	5.37	73.94	89.89	89.93	1.41	1x4x5
PCPDT-TTQ ($C_{54}S_{10}N_8H_{28}$)	24.12	24.48	8.01	6.95	115.48	90.0	90.0	1.50	1x3x4
PCPDT-TQ ($C_{56}S_6N_8H_{36}$)	24.29	8.09	13.97	11.01	103.71	103.74	97.18	1.46	3x2x2
PCPDT-BBT ($C_{48}S_8N_8H_{24}$)	24.26	9.02	12.63	9.93	92.03	116.24	91.47	1.59	3x2x3

Table S2: Electronic eigenstates (eV) and electron occupancies in valence and conduction band along various k-points in PCPDT-TQ computed using HSE06 method.

PCPDT-TQ					
		Valence Band		Conduction Band	
NKPTS	k-points	Energies	Occupancy	Energies	Occupancy
1	0.00000	-0.155175	0.99999	0.218112	0.00000
2	0.10179	-0.533547	1.00000	0.455730	0.00000
3	0.20358	-0.610803	1.00000	0.416906	0.00000
4	0.30538	-0.048322	0.91415	0.069746	0.02426
5	0.40717	-0.169290	1.00000	0.448957	0.00000
6	0.40717	-0.569562	1.00000	0.222252	0.00000
7	0.46616	-0.167227	1.00000	0.221640	0.00000
8	0.52514	-0.162237	1.00000	0.220172	0.00000
9	0.58413	-0.157246	1.00000	0.218717	0.00000
10	0.64312	-0.155175	0.99999	0.218112	0.00000
11	0.64312	-0.155175	0.99999	0.218112	0.00000
12	0.71953	-0.252590	1.00000	0.274023	0.00000
13	0.79594	-0.462167	1.00000	0.367572	0.00000
14	0.87236	-0.714602	1.00000	0.446298	0.00000
15	0.94877	-0.053905	1.00000	0.482241	0.00000
16	0.94877	-0.972562	0.93633	-0.015411	0.66850
17	1.07198	-0.515359	1.00000	0.349057	0.00000
18	1.19519	-0.580288	1.00000	0.550130	0.00000
19	1.31840	-0.312517	1.00000	0.355356	0.00000
20	1.44162	-0.155175	0.99999	0.218112	0.00000
21	1.44162	-0.155175	0.99999	0.218112	0.00000
22	1.52422	-0.253536	1.00000	0.272010	0.00000
23	1.60682	-0.464764	1.00000	0.360155	0.00000
24	1.68943	-0.717957	1.00000	0.431971	0.00000
25	1.77203	-0.046396	1.00000	0.463445	0.00000
26	1.77203	-0.976843	0.90529	-0.015369	0.66810
27	1.88107	-0.513585	1.00000	0.353501	0.00000
28	1.99010	-0.581472	1.00000	0.555003	0.00000
29	2.09914	-0.309905	1.00000	0.355612	0.00000
30	2.20818	-0.155175	0.99999	0.218112	0.00000
31	2.20818	-0.155175	0.99999	0.218112	0.00000
32	2.31557	-0.532514	1.00000	0.453721	0.00000
33	2.42296	-0.612653	1.00000	0.416415	0.00000
34	2.53036	-0.048454	0.91473	0.068561	0.02624
35	2.63775	-0.566941	1.00000	0.444709	0.000000

Table S3: Electronic eigenstates (eV) and electron occupancies in valence and conduction band along various *k*-points in PCPDT-BBT computed using HSE06 method.

PCPDT-BBT					
		Valence Band		Conduction Band	
NKPTS	k-points	Energies	Occupancy	Energies	Occupancy
1	0.00000	0.070886	0.02248	0.419819	0.00000
2	0.09715	-0.154835	0.99999	0.369137	0.00000
3	0.19429	-0.600328	1.00000	0.216960	0.00000
4	0.29144	-0.744150	1.00000	-0.021326	0.72681
5	0.38858	0.057371	1.00000	-0.222148	1.00000
6	0.38858	-0.523277	0.05233	0.410371	0.00000
7	0.45088	0.059564	0.04602	0.411696	0.00000
8	0.51317	0.064540	0.03397	0.414975	0.00000
9	0.57546	0.069117	0.0253	0.418367	0.00000
10	0.63776	0.070886	0.02248	0.419819	0.00000
11	0.63776	0.070886	0.02248	0.419819	0.00000
12	0.72603	0.025730	0.23338	0.427343	0.00000
13	0.81431	-0.094584	0.99627	0.448331	0.00000
14	0.90259	-0.269501	1.00000	0.477172	0.00000
15	0.99086	-1.189682	1.00000	0.499544	0.00000
16	0.99086	-0.485838	1.00000	0.445510	0.00000
17	1.10700	-0.576846	1.00000	0.112739	0.00071
18	1.22313	-0.497748	1.00000	0.048066	0.08700
19	1.33927	-0.271090	1.00000	0.328017	0.00000
20	1.45541	0.070886	0.02248	0.419819	0.00000
21	1.45541	0.070886	0.02248	0.419819	0.00000
22	1.56075	0.023958	0.24901	0.425870	0.00000
23	1.66610	-0.100380	0.99774	0.443554	0.00000
24	1.77145	-0.279169	1.00000	0.469826	0.00000
25	1.87680	-1.196277	1.00000	0.492800	0.00000
26	1.87680	-0.496987	1.00000	0.440332	0.00000
27	1.97490	-0.582804	1.00000	0.111315	0.00082
28	2.07299	-0.496178	1.00000	0.047397	0.09003
29	2.17109	-0.270321	1.00000	0.329907	0.00000
30	2.26918	0.070886	0.02248	0.419819	0.00000
31	2.26918	0.070886	0.02248	0.419819	0.00000
32	2.38214	-0.155990	0.99999	0.370633	0.00000
33	2.49509	-0.601150	1.00000	0.225888	0.00000
34	2.60805	-0.722245	1.00000	-0.007126	0.57987
35	2.72100	-0.480893	1.00000	-0.221307	1.00000

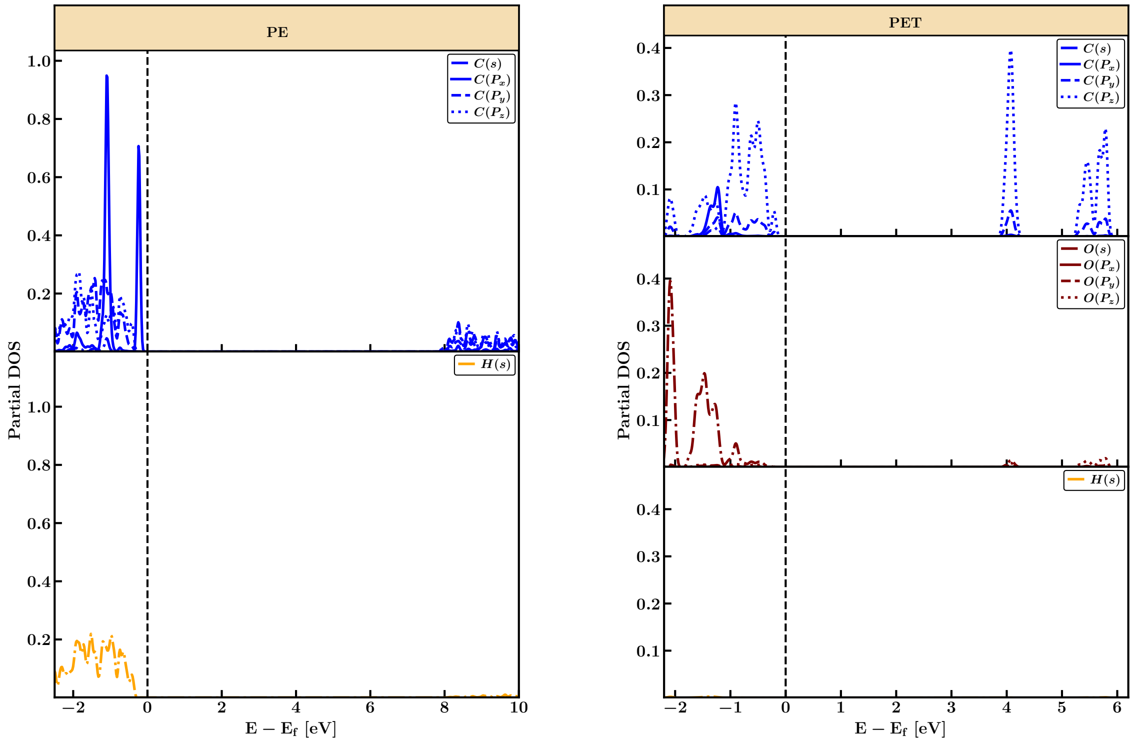

Figure S1: Partial density of states of PE and PET computed using DFT. The relative contributions of the individual orbitals (s, P$_x$, P$_y$ and P$_z$) of the C, O and H atoms are shown.

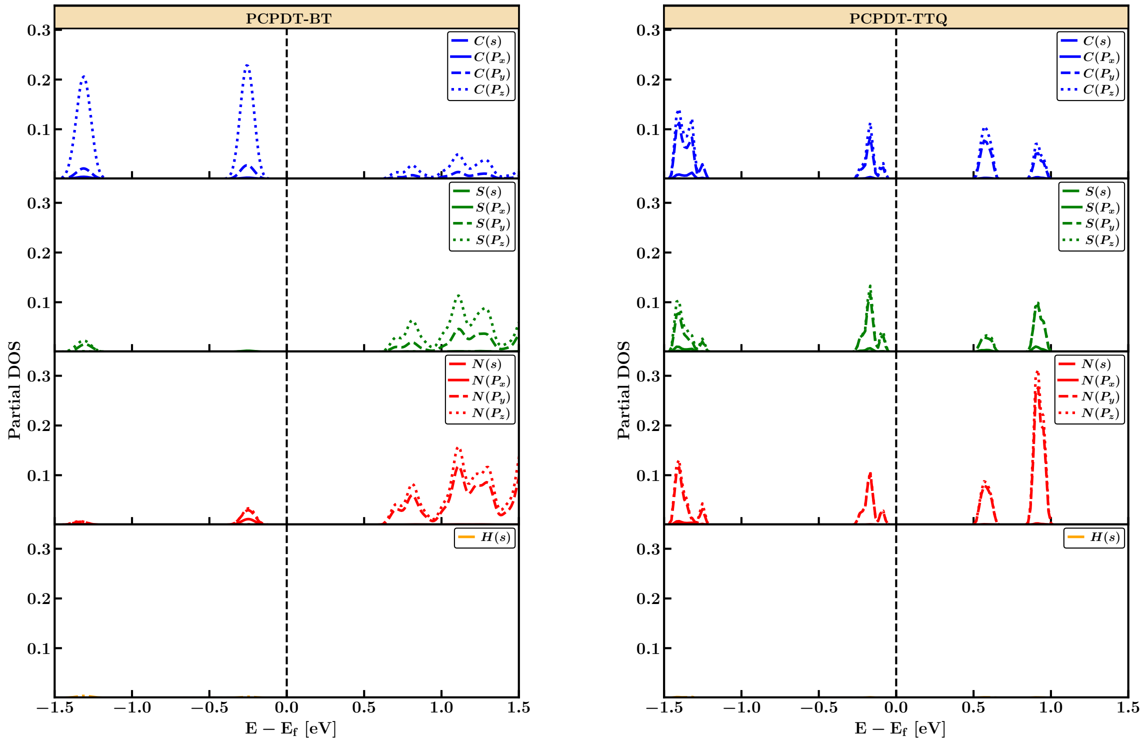

Figure S2: Partial density of states of PCPDT-BT and PCPDT-TTQ computed using DFT. The relative contributions of the individual orbitals (s, P_x, P_y and P_z) of the C, S, N, O and H atoms are shown.

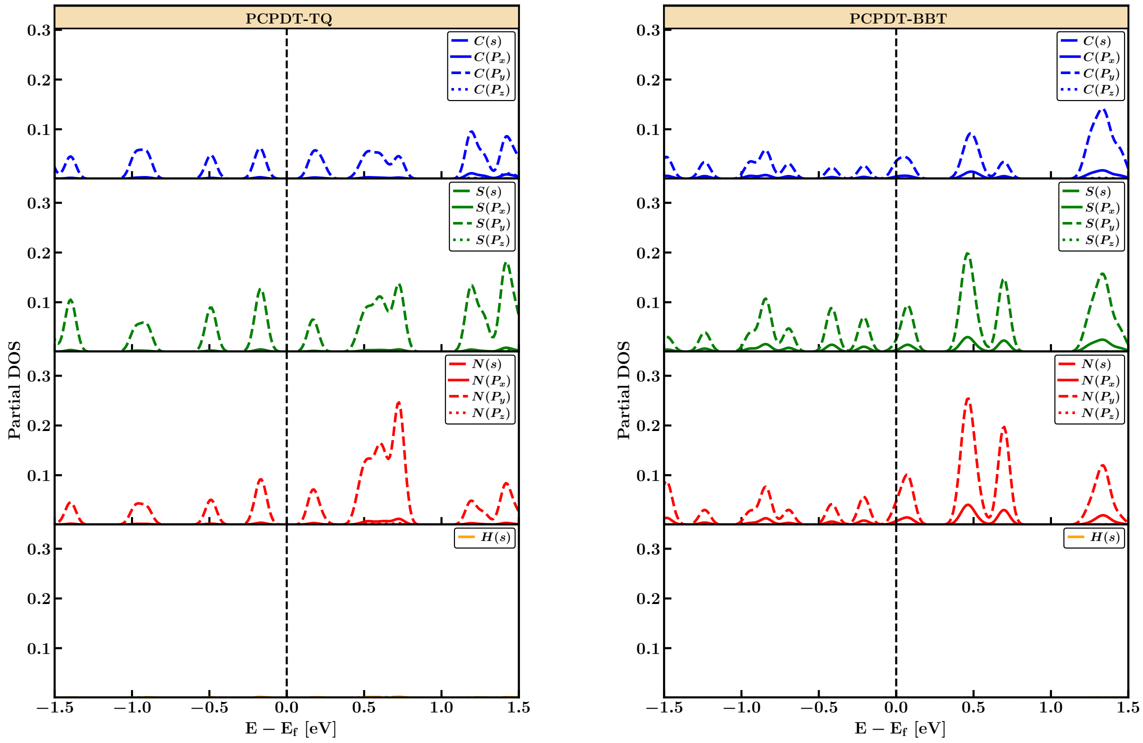

Figure S3: Partial density of states of PCPDT-TQ and PCPDT-BBT computed using DFT. The relative contributions of the individual orbitals (s, P_x, P_y and P_z) of the C, S, N, O and H atoms are shown.

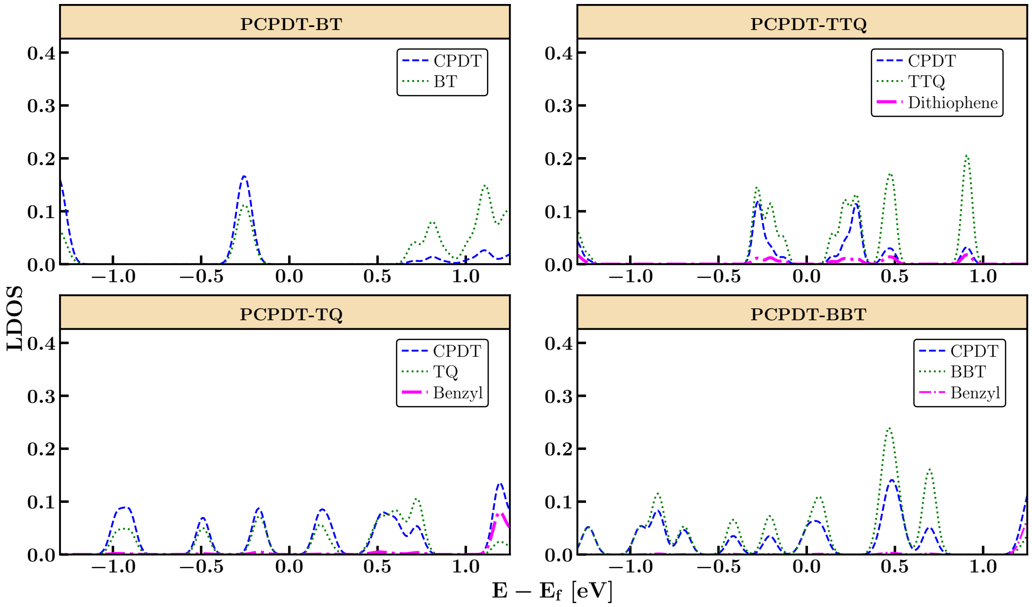

Figure S4: Local density of states (LDOS) of PCPDT-BT, PCPDT-TTQ, PCPDT-TQ and PCPDT-BBT computed using DFT. The relative contributions of the donor, acceptor and substitution units are shown.

Table S4: The band gap computed at Γ-point using G_0W_0, HSE06 and PBE methods. For PCPDT-TTQ, the band gap at G_0W_0 and PBE are computed with shifted k-point (0.2 -0.1 -0.2). Please see Methodology section in main article for more details.

	Electronic Gap at Γ-point (eV)		
	G_0W_0	HSE06	PBE
PE	10.286	9.185	7.682
PET	6.044	4.542	3.534
PCPDT-BT	1.713	1.188	0.650
PCPDT-TTQ	1.144	0.757	0.429
PCPDT-TQ	-	0.373	0.232
PCPDT-BBT	-	-	0.172

Table S5: Macroscopic static dielectric constant (ϵ) computed using density functional perturbation theory (DFPT) and BSE for various polymers. Known experimental dielectric constant values are also shown.[S2–S4]

Static Dielectric Constant									
	DFPT				BSE				Expt.
	ϵ_{xx}	ϵ_{yy}	ϵ_{zz}	ϵ	ϵ_{xx}	ϵ_{yy}	ϵ_{zz}	ϵ	ϵ
PE	2.85	2.88	2.89	2.87	1.75	1.80	1.98	1.84	2.25
PET	3.08	1.93	2.06	2.36	2.04	1.51	1.14	1.56	∼3.5
PCPDT-BT	31.16	2.94	3.11	12.40	23.51	1.21	1.73	8.82	3.6
PCPDT-TTQ	19.17	4.00	3.79	8.99	12.29	2.16	2.01	5.49	-
PCPDT-TQ	37.72	2.68	3.48	14.62	26.80	1.68	2.06	10.18	-
PCPDT-BBT	89.57	3.57	2.97	32.04	37.69	2.10	1.39	13.73	-

Table S6: The number of electrons, k-points, and empty levels for various polymers considered in G_0W_0 and DFPT calculations.

	No. of electrons	No. of k-points	No. of empty levels	
			G_0W_0	DFPT
PE	24	84	56	56
PET	72	32	168	48
PCPDT-BT	212	6	428	188
PCPDT-TTQ	344	16	696	296
PCPDT-TQ	336	8	664	304
PCPDT-BBT	304	6	616	216

Table S7: Macroscopic static dielectric constant (ϵ) computed using DFPT for PCPDT-TTQ, PCPDT-TQ and PCPDT-BBT with Γ-centered and the Monkhorst-Pack scheme. The shifted k-grid in the Monkhorst-pack is also shown.

	Γ- Centered		Monkhorst-Pack Scheme	
	k-grid	ϵ	k-grid	ϵ
PCPDT-BT	1x3x2	12.40	1x2x2<br>0.2 0.3 0.1	11.55
PCPDT-TTQ	1x4x4<br>0.2 0.3 0.1	9.38	1x2x2<br>0.2 0.3 0.1	8.99
PCPDT-TQ	1x2x4	14.62	1x2x4<br>0.1 0.3 0.2<br>2x2x7<br>0.1 0.3 0.2	11.79<br>12.91
PCPDT-BBT	1x2x3	32.04	1x2x2<br>0.1 0.3 0.2	32.79

Co-ordinates of polymers optimized using PBE method including spin-orbit coupling and van der Waals interactions.

Optimized coordinates of PE

C H
4 8
Direct

0.5000000000000000	0.7939120855990751	0.9376661365629175
0.0000000000000000	0.7060859632823124	0.0629998974370807
0.0000000000000000	0.2060879144009249	0.4376661365629175
0.5000000000000000	0.2939140367176876	0.5629998974370807
0.5000000000000000	0.9553966668499640	0.9702888190631285
0.5000000000000000	0.7718434030368897	0.7038105552198175
0.0000000000000000	0.5446023920314147	0.0303791919368734
0.0000000000000000	0.7281567218444920	0.2968554027801815
0.0000000000000000	0.2281565969631103	0.2038105552198175
0.0000000000000000	0.0446033331500360	0.4702888190631285
0.5000000000000000	0.2718432781555080	0.7968554027801815
0.5000000000000000	0.4553976079685853	0.5303791919368734

Optimized coordinates of PET

C H O
10 8 4
Direct

0.6478146033640897	0.0692158382322745	0.2566720130611486
0.5155850061096245	0.1091193649269471	0.1433406459202970
0.4495072493452312	0.9095004273009977	0.1967723388229530
0.5160851398070605	0.6716291989554080	0.3661861130218824
0.6483187459171944	0.6317265687759601	0.4794885631861305
0.7143538474136406	0.8313650387963634	0.4262789541789616
0.8531238069651010	0.8006073525557511	0.5534508195879582
0.3106692618267957	0.9402390696452372	0.0699625494968430
0.1049304802297044	0.2306666597762330	0.8629379330651048
0.0587189681258522	0.5103022783426923	0.7613719375943901
0.7026323056467660	0.2201863149194665	0.2129656909315543
0.4625835452122047	0.2936408911570254	0.0123278473273132
0.4613156292721925	0.5206214724517224	0.4097181279886968
0.7013600310838584	0.4471702341387456	0.6103195383058448
0.1168171350370173	0.1442416300193727	0.6364018860229095
0.0274738250035682	0.1509088388613833	0.0826027090901036
0.1362428293224269	0.5901116784509455	0.5419704791512316
0.0466494712635637	0.5967486336326502	0.9879895271547667
0.9014153090792760	0.9726566811208173	0.5742209414185950
0.2623703632130159	0.7681792376363603	0.0492445479462944
0.9199386075546911	0.5593791930480663	0.6444934022522162
0.2438139259239520	0.1814924653580690	0.9792795059653869

Optimized coordinates of PCPDT-BT

C S N H

34 6 4 20

Direct

0.3359222438299838	0.1571322238739867	0.9055967370988682
0.2887480367790758	0.0190156934688730	0.0410141603504428
0.3062382116918769	0.8221782768767838	0.2153398856350464
0.3655478705922732	0.8220661219331049	0.2152177294032853
0.3830580491784374	0.0188419395598203	0.0408348610664291

0.2046169101810236	0.8512883978417847	0.1544241600762817
0.2323843117090121	0.0358076586441882	0.0071874799939451
0.4672129296162524	0.8511434680409025	0.1542815807702240
0.4394397754873296	0.0356177112190537	0.0069821029332644
0.3358912606616542	0.3941893768242863	0.9280665069016365
0.3360069165467863	0.1538814716724133	0.6201209621778858
0.1460256209040054	0.8194743600585497	0.1506503955238756
0.1155508736063666	0.9367418366362301	0.9259926382059049
0.1147683273972504	0.6912309950135551	0.3542720626904767
0.0564319786919683	0.9367774759999392	0.9259373683926029
0.0570115463303225	0.6912365956644138	0.3542089754509306
0.0258406229405921	0.8194581501433120	0.1505848450547305
0.8359222438299838	0.1571322238739867	0.9055967370988682
0.7887480367790758	0.0190156934688730	0.0410141603504428
0.8062382116918769	0.8221782768767838	0.2153398856350464
0.8655478705922732	0.8220661219331049	0.2152177294032853
0.8830580491784374	0.0188419395598203	0.0408348610664291
0.7046169101810236	0.8512883978417847	0.1544241600762817
0.7323843117090121	0.0358076586441882	0.0071874799939451
0.9672129296162524	0.8511434680409025	0.1542815807702240
0.9394397754873296	0.0356177112190537	0.0069821029332644
0.8358912606616542	0.3941893768242863	0.9280665069016365
0.8360069165467863	0.1538814716724133	0.6201209621778858
0.6460256209040054	0.8194743600585497	0.1506503955238756
0.6155508736063666	0.9367418366362301	0.9259926382059049
0.6147683273972433	0.6912309950135551	0.3542720626904767
0.5564319786919683	0.9367774759999392	0.9259373683926029

0.5570115463303225	0.6912365956644138	0.3542089754509306
0.5258406229405921	0.8194581501433120	0.1505848450547305
0.2506813121897125	0.6574958645540789	0.3420358481732748
0.4211151324843740	0.6573617535932286	0.3418925199341203
0.0861148428881577	0.1512935042969730	0.5088145684364846
0.7506813121897125	0.6574958645540789	0.3420358481732748
0.9211151324843740	0.6573617535932286	0.3418925199341203
0.5861148428881577	0.1512935042969730	0.5088145684364846
0.1370422441027230	0.0551393518801859	0.6975875131786822
0.0350797983014388	0.0552058286382433	0.6975010506633410
0.6370422441027230	0.0551393518801859	0.6975875131786822
0.5350797983014388	0.0552058286382433	0.6975010506633410
0.2116109594258759	0.1746173451789943	0.8764026478152473
0.4602139978046083	0.1744741834272716	0.8762112767265933
0.3722979309323549	0.4838470067897447	0.8352419005459240
0.2995371407104699	0.4841223609269250	0.8352421120904339
0.3358246284568622	0.3963580954815811	0.1305790808807643
0.2994324206368475	0.2365579136477223	0.5208145405195168
0.3726057762473189	0.2364675309228517	0.5206593434268854
0.3360514281031897	0.9837941332413109	0.6057727124725005
0.1356486028040678	0.5986194795310738	0.5302872986920661
0.0360477282707876	0.5985635194185903	0.5302456347183337
0.7116109594258759	0.1746173451789943	0.8764026478152473
0.9602139978046154	0.1744741834272716	0.8762112767265933
0.8722979309323549	0.4838470067897447	0.8352419005459240
0.7995371407104699	0.4841223609269250	0.8352421120904339
0.8358246284568551	0.3963580954815811	0.1305790808807643

0.7994324206368546	0.2365579136477223	0.5208145405195168
0.8726057762473189	0.2364675309228517	0.5206593434268854
0.8360514281031897	0.9837941332413109	0.6057727124725005
0.6356486028040678	0.5986194795310738	0.5302872986920661
0.5360477282707876	0.5985635194185903	0.5302456347183337

Optimized coordinates of PCPDT-TTQ

C S N H

54 10 8 28

Direct

0.2547986926451458	0.0138777278199811	0.9723123559081657
0.2817723802934751	0.1198649693989751	0.8825178344547027
0.3377822732452671	0.1178741792169617	0.8995600645750201
0.3566944026430363	0.0133366830427306	0.0071097885055877
0.5170654803544679	0.0493298695389512	0.9938818708019994
0.4876469174154749	0.1400244893672138	0.8915833901685346
0.4319606037934705	0.1295937611163822	0.9081299007173342
0.4148750583650838	0.0233532499716489	0.0157248487654584
0.3843353140608201	0.2032075594480176	0.8295272764847681
0.0750037971778426	0.9594929951761344	0.9915523601805702
0.1015229897252041	0.0466093213385790	0.8749303238808039
0.1605754476716612	0.0580201053764497	0.8613351105225746
0.1972004809134944	0.9890950269914640	0.9730478342471258
0.1709100392876621	0.8931926406363075	0.0810473433544274
0.1122557435883849	0.8726635265523086	0.0831375655865543
0.1237674969378517	0.6912052547844709	0.2706645252451381
0.1820800575027377	0.7154260572469298	0.2734266410530211

0.2215374677525048	0.6359889859517125	0.3684675624878793
0.2736433255929356	0.7041903922658079	0.4386604708190660
0.3040164743925828	0.5928731979836712	0.5118837918903338
0.2751969268088459	0.4384240233085848	0.4931294088580458
0.0925133455016578	0.5925677321249054	0.3700213243026482
0.0962461895456954	0.5953420149571045	0.5699234172367369
0.0510350700523858	0.5105452238444173	0.6202071522694439
0.0140938509068675	0.4379337635820661	0.4550503875019487
0.3816924023463173	0.4166375734139791	0.9407400427048742
0.3872690375523220	0.1330590750131435	0.5863050381668202
0.7548027326245261	0.9860883149975734	0.0271147598301908
0.7817413568825771	0.8799448515781663	0.1167359629298730
0.8377538822371733	0.8822921878348069	0.1000592871380164
0.8566419438246058	0.9871972930409214	0.9929108313006907
0.0169870535579548	0.9512816917127722	0.0057586926508293
0.9876102363532340	0.8600355937403421	0.1075824048191052
0.9319283549896085	0.8708318647892526	0.0915798935541403
0.9148166110459997	0.9775743896913553	0.9846375151175621
0.8843021258876789	0.7971868793089740	0.1703020494989147
0.5750733739623186	0.0411630729661212	0.0083507666247016
0.6015524970153692	0.9538390170141255	0.1247443932838408
0.6605982509367507	0.9422521447094780	0.1382018486629306
0.6972398239688573	0.0111519950982668	0.0265858823256906
0.6709820021187127	0.1073113553742999	0.9189036911183024
0.6123530445134691	0.1280230710095580	0.9170057033336505
0.6238285449018832	0.3093710353601438	0.7295749422871012
0.6821420700489682	0.2850958080276058	0.7265792032695089

0.7214985105982024	0.3642838502215255	0.6307329454496866
0.7734946691964382	0.2958703132810285	0.5592727227462575
0.8037172732456455	0.4067806916043679	0.4849322508617391
0.7749431902013484	0.5614593429666144	0.5043848904315738
0.5925427492654194	0.4077970475128012	0.6300930138037728
0.5962120489210818	0.4048767115965859	0.4301036979483825
0.5509941129949212	0.4895364935593065	0.3797570393408876
0.5140830648210937	0.5620556443602567	0.5448778338846125
0.8817141135857085	0.5837411638776899	0.0596389563082198
0.8873284574366735	0.8676442132025457	0.4135886474212427
0.3028400956710584	0.9155418695975825	0.0846395271062690
0.4704808846346253	0.9457292395793715	0.1080119377078148
0.1204046160921948	0.1877353946544460	0.6408466860733526
0.2115199947854762	0.4307089951700860	0.3907829213357630
0.0332333548765078	0.4794004362395228	0.2432607598260930
0.8028094191958388	0.0846950168308425	0.9150728040120342
0.9703914799811599	0.0553482758448496	0.8922725015226476
0.6203726328349575	0.8123502724579836	0.3584985940426080
0.7114869549594047	0.5696830730333815	0.6086059486560913
0.5332513587210599	0.5207794260081968	0.7567760511987984
0.1754832635496797	0.1354884779429568	0.7312068836530585
0.0745563644256748	0.1172220632002237	0.7579148230412542
0.0922767545776040	0.7668515301012775	0.1733695177572088
0.2030027255717783	0.8146421854857238	0.1776257703777944
0.6754937314238489	0.8645510946435451	0.2681427690725542
0.5745719932534001	0.8830880612836793	0.2415854234416841
0.5923485939321296	0.2339395275502696	0.8270593138840852

0.7030754373784234	0.1858299018658940	0.8222742204061362
0.2589762429379832	0.1919783411867542	0.8074961060603840
0.5087796355505105	0.2105928478622374	0.8111594930854906
0.2880463137388460	0.8327001768440070	0.4391860461370101
0.3449218124132187	0.6259783646824602	0.5792761157177750
0.1299550495364912	0.6601233405404727	0.6778552077876085
0.0455770392107411	0.5055222692039436	0.7729103464022558
0.3448750628365218	0.4667431023456885	0.8915529809550051
0.4177539164769613	0.4763728709234130	0.8995078144707591
0.3508022964864423	0.1723975113862366	0.5239534090325577
0.3906148296745826	0.9814943916796466	0.5070991484760228
0.7589048379325760	0.8076227343322060	0.1914514086733945
0.0087952872735357	0.7891791669870685	0.1874976528915582
0.7879347776662371	0.1673749290136257	0.5586068568047509
0.8445586045790563	0.3734870298528179	0.4170278345112095
0.6299050234950130	0.3402471446993403	0.3221753168415660
0.5455183210135957	0.4945148981764831	0.2270585740271258
0.8449214675809671	0.5336423635811585	0.1089844486222447
0.9177881295512051	0.5245157464776042	0.1014152743924015
0.8509052858846644	0.8284099632844857	0.4763315791848513
0.8907252888966184	0.0191935886775241	0.4925999929544247
0.8802344522001277	0.5330381618370907	0.8857487272122455
0.9232806674145948	0.8097149992864630	0.4576509129187158
0.4231767436936806	0.1911543516650980	0.5421259410495409
0.3801557063224763	0.4669736043926704	0.1145799045258542
0.2874955051463388	0.3291244466827905	0.5366592536755093
0.9771673136708827	0.3596634252768354	0.4465110911871548

0.7872397616601745	0.6706591812053304	0.4606140429344876
0.4771576100816972	0.6401707685213083	0.5532706097217996

Optimized coordinates of PCPDT-TQ

C S N H

56 6 8 36

Direct

0.7297832930292714	0.9724239331672990	0.2151719711183659
0.7618701182185248	0.8802148883430121	0.2384885193499855
0.9323519061950236	0.8821449719238075	0.3006561788469284
0.0390545241068949	0.9778020807838530	0.3205431586086362
0.5096392384430501	0.9552069814174970	0.4823702612805576
0.3829720486159971	0.8677083080444490	0.4569923464659169
0.2177759276300790	0.8772496655957198	0.3972160637126834
0.2114250795452222	0.9738980766053444	0.3764569131385542
0.0411510033638862	0.8165400065667896	0.3528154987863701
0.5443777647259154	0.5981372013543336	0.3284640849562521
0.5802480429098651	0.5217412314279528	0.3869755614891162
0.7502094561966857	0.5078370145458777	0.4303108833860847
0.8842520869795862	0.5729272165633716	0.4156880991313514
0.8552235405724815	0.6548623322540337	0.3643154012734300
0.6818624795749031	0.6646361668874547	0.3190990427972835
0.0096239183140128	0.7246261138010652	0.3746387871602437
0.3598592461746435	0.6068045716794899	0.2743734198527150
0.7876488087056899	0.4261859622190229	0.4934961890371383
0.2165236964157629	0.0342623615086382	0.0457046176607250
0.2406366223966572	0.9385158040358306	0.0655927867459738

0.4086942978868962	0.9159094528441756	0.1189931108478675
0.5650932910648905	0.9906580711842352	0.1588379008033982
0.5405388807796569	0.0869570335103234	0.1401017452133217
0.3740841308346745	0.1094501869249598	0.0885765775744716
0.5092455360695212	0.2727598856249074	0.1119692446320073
0.6747822758074236	0.2496609479732541	0.1589617349955503
0.8433245068997550	0.3213780329293954	0.1910592140757501
0.4790676947173722	0.3720594857273198	0.0910460401227482
0.2549849714267722	0.0314162173701433	0.7215874749175484
0.3699701574155014	0.1227433800957769	0.7419237754606200
0.5409327214860085	0.1206318482474558	0.7962974223020822
0.5609819575981376	0.0264605487938780	0.8221259051608101
0.0509965762467246	0.0507216050641688	0.9864985371552564
0.0063159153946373	0.1383940537903072	0.9532363351313577
0.8317289906442014	0.1280847980329085	0.8948239237883229
0.7361141454106672	0.0311203989934867	0.8812999134670321
0.7117279792477689	0.1883555359840585	0.8406353407429137
0.4021682838342500	0.3643602582299437	0.6103321597895217
0.3881209429439920	0.4542657613218637	0.6928112201961767
0.4973701679595806	0.4924982540340466	0.8202152479992222
0.6256090811226755	0.4401262280143285	0.8630018787344227
0.6338434225329408	0.3456047974683045	0.7862660518711948
0.5225002023052028	0.3096296040256448	0.6596697574299952
0.7536902872592179	0.2858078116961309	0.8406160488366297
0.2904131606122533	0.3248152849151609	0.4719114937029545
0.4702069788721346	0.5842088508106045	0.9116111840758165
0.6897145582348472	0.9710961786246983	0.5426221179487385

0.8007265555818179	0.0642619518421128	0.5592806002264368
0.9858786124456245	0.0860764662178752	0.6206695006064535
0.0739729015355906	0.0123682422686286	0.6647770256318424
0.9623858418515994	0.9193932890472851	0.6496590925713477
0.7768230976280179	0.9010300904448485	0.5993356880155503
0.7616798080764369	0.7557258371216733	0.6672884315596264
0.9456292744756993	0.7661666573044030	0.6979729293296018
0.0365930729790946	0.6882901892537205	0.7419887624485071
0.6533186382647784	0.6764372939680214	0.7004753999173872
0.9244678495237721	0.0636249916753968	0.2689829223770630
0.4112731627430648	0.0506608308278587	0.4275158803055277
0.1927875750546875	0.7679956633581000	0.0762428147732166
0.3687932652260244	0.9415783392568429	0.7755602982780445
0.8632388738042849	0.9543238324834959	0.9402635952098635
0.9075192017720966	0.2338264426986285	0.5667366730779548
0.3971594798695648	0.8208940711388450	0.1279700161367003
0.1093365744525343	0.8603836604858444	0.0371600453318095
0.3661907647208480	0.2035146646083916	0.0802343049459395
0.6840619763604536	0.1585563181143925	0.1739668893896820
0.0593701271484335	0.1804578883298262	0.6305225035369091
0.7422033274786486	0.1421941147605352	0.5236612794453492
0.6827257881052660	0.8192517922197098	0.6136678646076419
0.0398077754709192	0.8476172258779968	0.6897919065848086
0.6545921788859346	0.8172390150854483	0.2056069583674613
0.4212606473661751	0.8034234706232084	0.4874691825318322
0.4736531011303029	0.4713625079016666	0.3978983840351233
0.0176885480645907	0.5629453845960413	0.4496743527225391

0.6522252206274644	0.7264921467197780	0.2797639103722673
0.3123647607314837	0.5665584412480271	0.1699627301007496
0.3465871405789258	0.6852319679832775	0.2839303697203377
0.2734249850574315	0.5733244528352444	0.3228492629526869
0.9253633453121353	0.4217968657831648	0.5131904071517823
0.7122782795852203	0.3524656432241571	0.4313943400917495
0.7535039260172454	0.4381568324322913	0.5862220642326577
0.1279671242741500	0.7013918402795696	0.4196860876951831
0.8864704615371224	0.3171854516723656	0.1027392137809713
0.9431386263826909	0.3001382342404995	0.2606859210830237
0.8336113481534753	0.3995294425721099	0.2309155632716156
0.4346907433062484	0.3638534740073425	0.9860737709483161
0.5937156933142731	0.4320246305036335	0.1330510969859233
0.3751986499209110	0.3949409091888327	0.1331052065525995
0.3216189544438137	0.1857759014175144	0.7148728355428418
0.1065972850156598	0.2039586800316044	0.9738037059646132
0.2908580516835002	0.4963864203516195	0.6572491067275053
0.7137352022201497	0.4700881509063919	0.9610004974203079
0.5318612854331661	0.2380696773508291	0.5980294706050344
0.2113729323167561	0.3784842741062633	0.4430757787070903
0.2019714945746998	0.2542303320706765	0.4584097202385635
0.3689597178842234	0.3077782984123871	0.4033773656736059
0.3766070121890408	0.5623373798451397	0.9630309795618501
0.4171177344111499	0.6368431080605461	0.8603533912096992
0.5903211210442052	0.6247695474345392	0.9865297090456977
0.8876722581991174	0.3248548229398409	0.8898918787701220
0.1770988769743198	0.7156606400610741	0.7733929297939923

0.0055918290963604	0.6181347725664494	0.6632042466203245
0.9954042378911865	0.6694221048090299	0.8229302258963997
0.6824920031191013	0.6919207548354009	0.8069790861813289
0.6784463321707506	0.6011371341071055	0.6636839746780083
0.5155254297959360	0.6762680355362676	0.6611707086849350

Optimized coordinates of PCPDT-BBT

C S N H

48 8 8 24

Direct

0.8868886475180489	0.0893178939541670	0.0466928421395352
0.7702817628694376	0.1149528153626278	0.1072436216938470
0.9213399689775770	0.9832353812651107	0.0159662922857251
0.6799234486081929	0.0342396118631001	0.1432047730857633
0.7168099604939258	0.9285102827458900	0.1142498118383060
0.8311923144945794	0.9029694549621112	0.0522099488704200
0.5662797089274818	0.0540919328843543	0.2022059632562545
0.5183713018062193	0.1538922319403255	0.2359171350776847
0.4063355503319173	0.1469526069977221	0.2964981395779418
0.3639632789316565	0.0376985611973950	0.3067173053094052
0.0416411813126700	0.0396990546776195	0.4657619974124927
0.0999485589939368	0.1418532433776747	0.4502249078345812
0.2153790707959260	0.1380250602356909	0.3945665713828461
0.2485459399139103	0.0323750512275112	0.3646883180015479
0.3131073431891380	0.2141682502982576	0.3540475285008782
0.5676888957798880	0.5038631804948039	0.2699742684620503
0.4872320409668234	0.5986360525976906	0.2629827833976677

0.3522160608688480	0.6037428155056617	0.2971288000830299
0.2945364787472400	0.5109052169506541	0.3338396241545070
0.3689414462179101	0.4134780771028730	0.3374046727264428
0.5080704776668910	0.4128701308505853	0.3086837031762713
0.2975834583277859	0.3204881296416531	0.3712265767917273
0.7140365501657442	0.4972914931878094	0.2359139893744242
0.2751519901461776	0.7075536624265126	0.2986753118965666
0.8749140800240269	0.9104313668331088	0.5357911844821999
0.7543053471620169	0.8853386077612058	0.5922134607044569
0.9245138269895676	0.0162575319525970	0.5197505785908660
0.6743881636686169	0.9663123775455205	0.6381720233980843
0.7263819390932795	0.0718955281681204	0.6242795421044534
0.8457390173397101	0.0969492199988196	0.5671485479952167
0.5577014961681641	0.9465189014612392	0.6939614918183139
0.4962903100985088	0.8464220245923926	0.7129296296115371
0.3860144694691172	0.8525148280135753	0.7754676996577103
0.3579117242214593	0.9616426825946931	0.8009059321581162
0.0344541720742413	0.9591792292249366	0.9580427173350330
0.0772878860695485	0.8568749923122141	0.9267599331507483
0.1933006829675818	0.8606079665814761	0.8715493228951416
0.2421870296589930	0.9663954078641694	0.8583286972818911
0.2843089142694879	0.7847495420448496	0.8238818978019395
0.5586372700359163	0.4990589019864231	0.7713801668467184
0.5016169871834535	0.4010374480727350	0.7973521391684599
0.3689224841573520	0.3936071347984793	0.8348790133715056
0.2917666528896206	0.4861288253448137	0.8439707266928238
0.3440117040742479	0.5858382582976276	0.8163704613046221

0.4779152706776557	0.5895692202882259	0.7802044042088667
0.2646137522585192	0.6785547918803587	0.8376477225565466
0.7045885126002389	0.5103577859373516	0.7369499038804577
0.3126173710220641	0.2887538676784231	0.8675155393889256
0.8863749843653466	0.2790320702465934	0.0715764152808944
0.7217980931352201	0.7391606794582017	0.0940635818109854
0.8500004925970472	0.7209281564052219	0.5375760760436208
0.7592359329267637	0.2611563585903482	0.6318617897639740
0.1367918878217367	0.9385552069177905	0.4071379409783304
0.4641654506735691	0.9488673161897623	0.2460370935916671
0.1446539445106509	0.0601565228329406	0.9152776514972487
0.4695603209408290	0.0512052564197347	0.7529063419077886
0.7598013468229681	0.2201078869438930	0.1251958193515321
0.9597116037530569	0.1759569909198788	0.0227121959001266
0.6481915108166518	0.8417766972515111	0.1428324716843008
0.8428937544451927	0.7977404663575456	0.0356345740687729
0.7301303972312283	0.7802982624443686	0.5971931074651806
0.9368916209529132	0.8235168435418601	0.5015673385311459
0.6694279475525988	0.1587648290524655	0.6644484464235205
0.8732368222984306	0.2021049467821854	0.5664016359455246
0.5692294517741558	0.2250863428469856	0.2113937763377720
0.0533403104909738	0.2128473307707424	0.4798355947776471
0.5306807690140829	0.6712980684194534	0.2327059142373074
0.1893468146666208	0.5133555994804908	0.3605588433779019
0.5740606689350614	0.3405789736473679	0.3206677605427402
0.7758208728592422	0.4226171140793156	0.2727139834839107
0.8049690874044586	0.5634695387537860	0.2911971544338101

0.6746999938560450	0.4998474428534934	0.1141921179029524
0.2549385388912029	0.7521145702082066	0.1994717900153233
0.3574006827037621	0.7577483919814156	0.3982710725189094
0.1560833583822614	0.6967314299779801	0.3025701065847102
0.2091873330717746	0.3394332284080477	0.4154721871460936
0.5375447634429307	0.7759244677514872	0.6767580786438145
0.0198741095265831	0.7860567158908296	0.9456467237088333
0.5629449822999817	0.3287076269711520	0.7918084087698460
0.1915250466463263	0.4819016215915184	0.8774169270925825
0.5250543078819874	0.6652512873102623	0.7642132397605650
0.7558642318009063	0.5924438167309916	0.7608899562081177
0.6681339879317036	0.4903041319094612	0.6173187147509225
0.8030699472068576	0.4578880303613104	0.8032020385784477
0.4175287888716284	0.2377666471489590	0.9218318719817375
0.2222376414845684	0.2463216911483883	0.7634863225661022
0.2527727597518563	0.2988285707848846	0.9420542862744483
0.1730404947260809	0.6596442975596304	0.8786881147698153

References

(S1) Mark, J. *Polymer Data Handbook*; Oxford University Press: Oxford, U.K., 2009.

(S2) Armin, A.; Stoltzfus, D. M.; Donaghey, J. E.; Clulow, A. J.; Nagiri, R. C. R.; Burn, P. L.; Gentle, I. R.; Meredith, P. Engineering dielectric constants in organic semiconductors. *J. Mater. Chem. C* **2017**, *5*, 3736–3747.

(S3) Sears, F. W.; Zemansky, M. W.; Young, H. D. *University Physics, 6th Ed.,*; Addison Wesley, 1982.

(S4) Konieczna, M.; Markiewicz, E.; Jurga, J. Dielectric properties of polyethylene terephthalate/polyphenylene sulfide/barium titanate nanocomposite for application in electronic industry. *Polymer Engineering & Science* **2010**, *50*, 1613–1619.
